# Adjuvant immunotherapy *versus* observation after neoadjuvant therapy and esophagectomy for esophageal cancer with residual pathologic disease: a systematic review and reconstructed individual participant data meta-analysis

**DOI:** 10.3389/fimmu.2026.1944032

**Published:** 2026-09-24

**Authors:** Hongzhi He, Hanlu Zhang, Haowen Lv, Wenping Wang

**Affiliations:** 1 Department of Thoracic Surgery, West China Hospital, Sichuan University, Chengdu, China; 2 West China School of Medicine, Sichuan University, Chengdu, China

**Keywords:** esophageal neoplasms, esophagectomy, adjuvant immunotherapy, meta-analysis, neoadjuvant therapy

## Abstract

**Background:**

The role of adjuvant immunotherapy after neoadjuvant therapy and esophagectomy remains uncertain. We examined its association with survival in populations composed predominantly of patients with residual disease across neoadjuvant strategies.

**Methods:**

This systematic review included comparative studies of postoperative immunotherapy *versus* observation or placebo. The majority of the intervention groups received adjuvant immune checkpoint inhibitor (ICI) monotherapy, while three cohorts included patients treated with concomitant postoperative chemotherapy. The reported hazard ratios (HRs) were synthesized using random-effects models and were estimated from reconstructed Kaplan–Meier data when unavailable. Reconstructed data were also used to generate the survival curves. Bayesian hierarchical models and sensitivity analyses assessed robustness.

**Results:**

A total of 13 studies, including two randomized trials and 11 retrospective cohorts, were included. The overall survival (OS) data came from 12 studies with 4,225 patients, while the disease-free or recurrence-free survival (DFS/RFS) data were from 11 studies with 2,363 patients. Postoperative immunotherapy was associated with longer OS [HR = 0.64, 95% confidence interval (CI) = 0.53–0.77] and DFS/RFS (HR = 0.74, 95%CI = 0.66–0.84). The associations were favorable after neoadjuvant chemoradiotherapy (NCRT), chemoimmunotherapy (NCIT), and chemotherapy (NCT). Interaction testing was significant for OS, but not for DFS/RFS. The paradigm estimates were not direct comparisons as the histology, geography, and study design differed across strategies. Sensitivity analyses addressing residual disease eligibility and postoperative treatment composition yielded similar average estimates. Analyses restricted to studies with aligned postoperative time origins remained directionally favorable, but were based on only a few studies. The Bayesian pooled estimates were concordant, although the predictive intervals crossed 1 for both outcomes. Certainty was low.

**Conclusions:**

Randomized evidence supports a DFS benefit from adjuvant immunotherapy after NCRT. However, a statistically significant randomized OS benefit has not been demonstrated. The associations after NCIT were favorable, but entirely observational. The favorable estimates after NCT were based on only two small retrospective studies, while mixed neoadjuvant treatment was represented by a single study with imprecise estimates. The esophageal squamous cell carcinoma (ESCC)-predominant and residual pathology findings were exploratory. Further randomized studies are needed to define the role of postoperative immunotherapy after NCIT or NCT and to determine whether pathologic features can inform patient selection.

**Systematic Review Registration:**

https://www.crd.york.ac.uk/PROSPERO/, identifier CRD420261384875.

## Introduction

Esophageal cancer remains one of the most common malignancies worldwide and is the sixth leading cause of cancer death (1). Neoadjuvant chemoradiotherapy (NCRT) followed by surgery improved survival in esophageal and junctional cancer in the CROSS trial, and subsequent randomized evidence further supports NCRT or neoadjuvant chemotherapy (NCT) as the major treatment options for locally advanced esophageal cancer (2–5). Nevertheless, pathologic residual disease after neoadjuvant therapy is common and identifies a population at high risk of recurrence, as pathologic complete response (pCR) is achieved in only approximately 29%–43.2% of patients after NCRT and in approximately 5% after NCT (2, 6–8).

The postoperative treatment landscape changed after CheckMate 577, in which adjuvant nivolumab doubled the median disease-free survival (DFS) compared with placebo after NCRT and R0 resection in patients with residual pathologic disease (9). Updated results showed numerically longer overall survival (OS) with nivolumab, although the randomized comparison was not statistically significant and the OS difference was less pronounced than the DFS benefit (10). Adjuvant nivolumab has since then been widely used after NCRT for such patients; however, new questions have emerged with newer preoperative paradigms. Neoadjuvant chemoimmunotherapy (NCIT) has been rapidly adopted in esophageal squamous cell carcinoma (ESCC), particularly in Asia, where high pathologic response rates and promising survival outcomes have made it an attractive alternative to NCRT (11–15). It remains unclear whether postoperative immunotherapy improves OS after NCRT to the same extent as DFS. Whether immunotherapy should be continued after surgery for patients with residual disease after preoperative programmed cell death protein 1 (PD-1) or programmed cell death ligand 1 (PD-L1) blockade also remains uncertain.

Comparative cohorts have reported variable survival outcomes after adjuvant immunotherapy, while real-world studies have extended CheckMate 577 to broader populations and examined whether a DFS benefit is accompanied by an OS benefit (16). Differences in the neoadjuvant treatment, histology, and study design, however, complicate interpretation and leave the postoperative role of immunotherapy uncertain.

Previous meta-analyses have addressed adjuvant therapy after neoadjuvant treatment and esophagectomy without focusing on a specific postoperative modality. Lee et al. assessed the association between adjuvant therapy and survival after curative resection, with subgroup analyses by nodal status (17). Wang et al. updated this evidence and mainly reported time-specific OS rates, with additional analyses of DFS/recurrence-free survival (RFS) and clinicopathologic subgroups (18). However, neither study was designed to investigate postoperative PD-1/PD-L1 blockade in patients with residual disease after neoadjuvant therapy and surgery. We therefore performed a systematic review and reconstructed individual patient data (IPD) meta-analysis of adjuvant immunotherapy *versus* observation or placebo, focusing on the OS, DFS/RFS, neoadjuvant treatment paradigm, pathologic residual risk, and certainty of evidence.

## Methods

### Study design and eligibility

This systematic review and meta-analysis was prospectively registered in PROSPERO (CRD420261384875) and conducted in accordance with Preferred Reporting Items for Systematic Reviews and Meta-Analyses (PRISMA) 2020 (19). The review examined the association between postoperative treatment containing a PD-1 or a PD-L1 immune checkpoint inhibitor (ICI) and survival after neoadjuvant therapy and curative-intent esophagectomy.

Eligible studies enrolled adults with ESCC, esophageal adenocarcinoma, or gastroesophageal junction adenocarcinoma treated with neoadjuvant therapy followed by esophagectomy. The majority of the analytic cohorts were restricted to patients with pathologic residual disease. Residual pathologic disease was defined according to the criteria used in each original study, most commonly as viable residual tumor in the resection specimen (ypT1–4 and/or ypN1–3) or as an incomplete pathologic response according to the study-specific tumor regression grading system. The cohort reported by Chen et al. was not formally restricted to residual disease. It was retained because only 16 of the 169 patients (9.5%) had ypT0 disease and the number of those with ypT0N0 was not reported separately. A sensitivity analysis excluded this cohort.

Eligible postoperative interventions contained a PD-1 or a PD-L1 inhibitor, administered alone or with chemotherapy. Comparators were observation or placebo. A study was eligible only when an ICI-containing postoperative strategy could be distinguished from a non-ICI comparator. Studies that combined immunotherapy, chemotherapy, or radiotherapy within a single undifferentiated adjuvant treatment group were excluded because the association with an ICI-containing strategy could not be estimated.

Randomized controlled trials and comparative retrospective cohort studies were eligible. Studies restricted to patients with pCR, single-arm studies, conference abstracts without extractable survival data, and studies without an appropriate non-ICI comparator were excluded. The primary outcome was OS. The secondary outcome was DFS or RFS, using the definitions reported in the original studies. DFS and RFS were pooled as recurrence-related time-to-event outcomes while retaining the definitions used in each study.

### Search strategy and data extraction

PubMed, Embase, Web of Science, and the Cochrane Library were searched from inception to 2 May 2026. The search terms combined esophageal cancer, neoadjuvant therapy, postoperative treatment, and immune checkpoint inhibition. The reference lists of eligible studies and relevant reviews were screened, and citation tracking was performed. The complete search strategy is provided in the **Supplementary Methods**.

Two reviewers (HH and HL) independently performed study selection and data extraction. Disagreements were resolved by consensus with a third reviewer (HZ). Extracted information included study design, histology, neoadjuvant treatment, postoperative regimen, use of concomitant chemotherapy, comparator, analysis population, follow-up, and survival outcomes. Information relevant to treatment timing included the time origin for survival analysis, the interval from surgery to treatment initiation, the handling of recurrence or death before treatment, and the use of landmark or time-dependent analyses.

Article-reported hazard ratios (HRs) and 95% confidence intervals (CIs) were used when they matched the eligible population, comparison, and outcome. When several estimates were available, the randomized, adjusted, matched, weighted, or subgroup-specific estimates closest to the review question were preferred. If no suitable HR was reported, an HR was estimated from reconstructed Kaplan–Meier individual participant data using a Cox model. Fixed-time survival proportions were not converted into HRs.

### Risk of bias, immortal time bias, and certainty of evidence

Two reviewers (WW and HZ) independently assessed risk of bias. Randomized trials were evaluated using the Cochrane Risk of Bias 2 tool (20), and observational studies were assessed using the Newcastle–Ottawa Scale. Certainty of evidence was evaluated using GRADE (Grading of Recommendations Assessment, Development, and Evaluation), considering risk of bias, inconsistency, indirectness, imprecision, and publication bias (21).

Potential immortal time (guarantee time) bias was assessed separately for each study and outcome. Matching, weighting, and conventional Cox adjustment were not considered sufficient to address immortal time bias unless the treatment timing or eligibility at a common time point was explicitly incorporated. Studies were classified according to whether they aligned the time origin between treatment groups, excluded relevant early events, used a landmark analysis, or modeled treatment as a time-dependent exposure.

Publication bias judgments considered funnel plot asymmetry, Egger regression, the number of studies, study design, and possible clinical explanations for asymmetry. A significant Egger test alone was not used as a criterion for certainty downgrading.

### Statistical analysis

Study-level log HRs were pooled using restricted maximum likelihood (REML) random-effects models with the metafor package in R version 4.6.0. Pooled results are reported as HRs with 95%CIs. Heterogeneity was assessed using Cochran’s *Q*, *I*^2^, and *τ*^2^. Prediction intervals were calculated when supported by the number of studies. They were not reported for small descriptive sensitivity sets because these intervals would be unstable. DerSimonian–Laird, Paule–Mandel, and Sidik–Jonkman estimators were examined in the sensitivity analyses of between-study heterogeneity. Knapp–Hartung adjustment was used as a small-sample check of uncertainty around the pooled estimates.

Neoadjuvant treatment was classified as NCRT, NCIT, NCT, or mixed neoadjuvant therapy (Mixed NAT). Mixed NAT referred to cohorts containing more than one neoadjuvant approach that could not be separated for analysis. Paradigm-specific estimates were treated as subgroup analyses rather than direct comparisons between neoadjuvant strategies. Pathologic residual patterns were examined in exploratory analyses.

The sensitivity analyses included leave-one-out analysis; exclusion of HRs reconstructed from the Kaplan–Meier curves; restriction to randomized, adjusted, matched, or weighted estimates; restriction to explicitly defined residual disease cohorts; exclusion of national database studies; and exclusion of influential or outlying studies. In a *post-hoc* sensitivity analysis, we excluded cohorts in which at least half of the ICI-treated group received concomitant postoperative chemotherapy or when the contribution of chemotherapy could not be separated. Immortal time bias was examined in descriptive analyses restricted to studies with an aligned time origin and in broader analyses that explicitly excluded early events or used landmark methods. These analyses were interpreted with caution when only a few studies were available.

Complementary Bayesian random-effects meta-analyses were performed using bayesmeta version 3.5. On the log-HR scale, the pooled mean effect was assigned a normal prior, *N N*(0, 1^2^), and the between-study heterogeneity was assigned a half-normal prior with a scale of 0.5. Posterior median HRs and 95% credible intervals (CrIs) were estimated for the pooled effects, together with 95% predictive intervals and the posterior probability that the pooled HR was less than 1. Prior sensitivity analyses used *N*(0, 2^2^) for the pooled effect and half-normal heterogeneity priors with scales of 0.25 and 1.0.

The Kaplan–Meier curves were digitized using Engauge Digitizer version 12.1, and individual participant data were reconstructed using IPDfromKM version 0.1.10 (22). When reconstructed data from multiple studies were combined, the Cox model was stratified by study. This allowed each study to have a different baseline hazard while estimating a common treatment effect. Conventional Cox models were used when an analysis contained only one study. The main analyses and those restricted to ESCC-predominant cohorts included NCIT, NCRT, and NCT studies. Mixed NAT was excluded because it could not be assigned to a single neoadjuvant paradigm. The combined reconstructed data were analyzed as a complementary survival analysis, and the resulting Cox estimate was not used in the meta-analysis. If an eligible study did not report a suitable HR, an HR estimated from that study’s reconstructed Kaplan–Meier data was used as its study effect estimate.

Funnel plots and Egger regression were used to assess small-study effects when at least 10 studies were available. All tests were two-sided, and a *p* < 0.05 was considered statistically significant.

ChatGPT5.5 assisted with the language editing, formatting, and review of the R code. All contents were verified by the authors, who take responsibility for the final manuscript.

## Results

### Study selection and characteristics

The PRISMA 2020 flow diagram summarizes the study selection (**Figure 1**). After removal of duplicates, 1,568 records were screened and 60 full-text articles were assessed. A total of 13 studies were included in the quantitative synthesis (9, 10, 16, 23–33). One full-text study was excluded because its postoperative treatment group combined immunotherapy, chemotherapy, and radiotherapy, precluding an ICI-specific comparison (34). The study characteristics and the treatment regimens are presented in **Table 1** and **Supplementary Table S1**.

**Figure 1:**
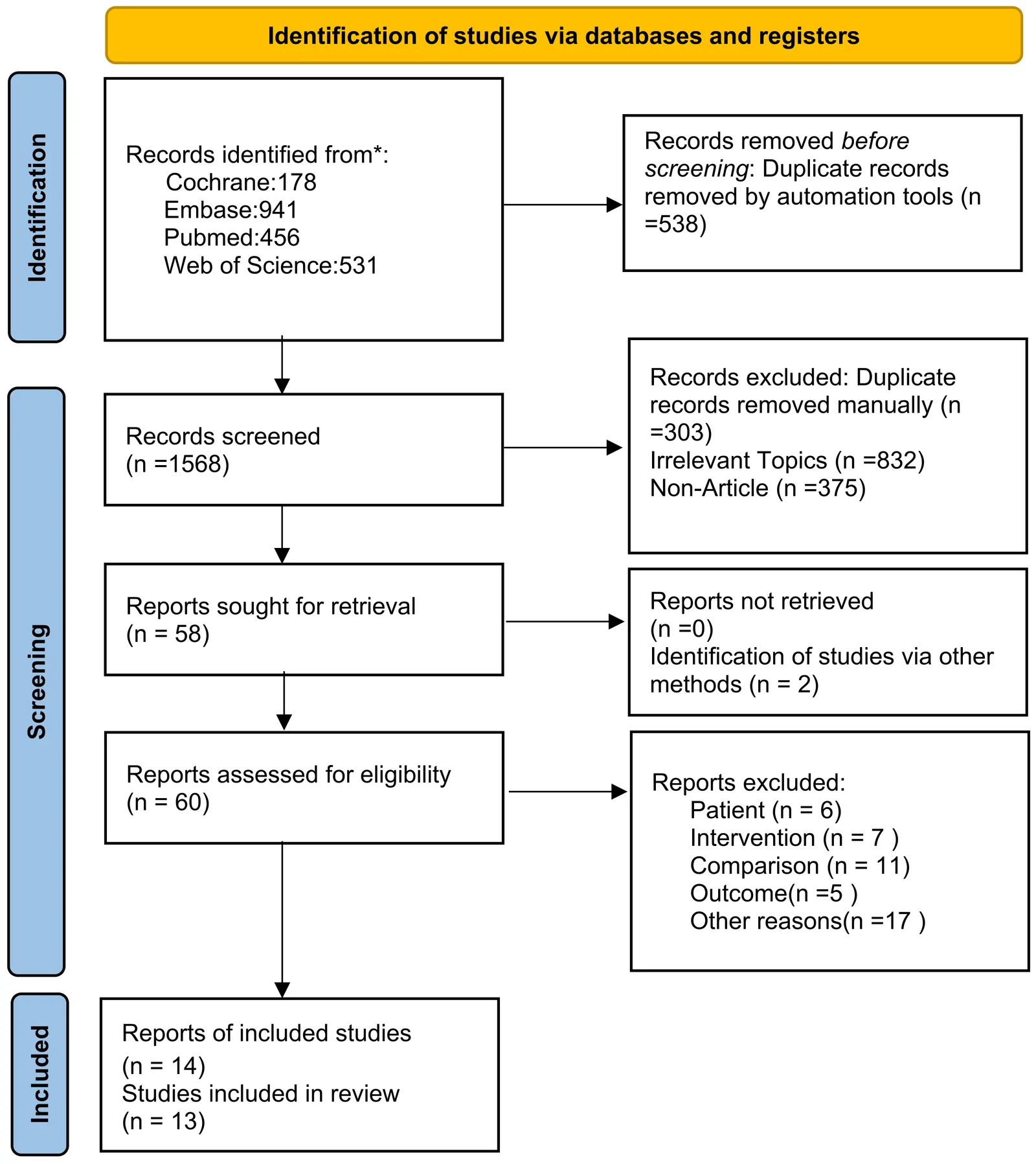
Preferred reporting items for systematic reviews and meta-analyses (PRISMA) 2020 flow diagram showing study selection.

**Table 1:** Characteristics of the included studies and meta-analysis populations.

Study	Design and data source	Neoadjuvant paradigm	Postoperative treatment in the intervention arm	Analysis population	OS AIT/control, *n*	DFS/RFS AIT/control, *n*	Comparator	Follow-up (months)
Chen et al., 2025 (24)	Retrospective multicenter cohort, China	NCIT	PD-1 inhibitor	Predominantly residual disease; ypT0 16/169 (9.5%); ypT0N0 NR	82/87	82/87	Ob	34.0, overall
Chen et al., 2026 (23)	Retrospective multicenter cohort, China	NCIT	PD-1 inhibitor	Non-pCR after NCIT and R0 resection	221/186	221/186	Ob	31.2 in both arms, survivors
Feng et al., 2024 (25)	Retrospective single-center cohort, China	NCIT	PD-1 inhibitor	Residual disease after NCIT and surgery	67/83	67/83	Ob	40.0, overall
Geng et al., 2026 (26)	Retrospective multicenter cohort, China	NCIT	ICI monotherapy or ICI plus chemotherapy	Non-pCR after NCIT and surgery	126/98	126/98	Ob	21.6, overall
Huang et al., 2026 (27)	Retrospective two-center cohort, China	NCIT	ICI monotherapy or ICI plus chemotherapy	Residual-risk subgroup	NR	86/43	Ob	25.8, overall
Kelly et al., 2021/2025 (9, 10)	Phase 3 randomized placebo-controlled trial, international	NCRT	Nivolumab monotherapy	Residual disease after NCRT and R0 resection	532/262	532/262	Pbo	78.3, final OS; 24.4, primary DFS
Koga et al., 2025 (28)	Retrospective two-center cohort, Japan	NCT	Nivolumab monotherapy	Non-pCR after NCT and surgery	15/38	15/38	Ob	27.5/15.2, by arm
Liu C et al., 2025 (29)	Retrospective single-center cohort, Taiwan	Mixed NAT	ICI monotherapy; agent not reported	Non-pCR after mixed NAT and surgery	23/68	21/62	Ob	25.0, overall
Park et al., 2022 (30)	Phase 2 randomized placebo-controlled trial, South Korea	NCRT	Durvalumab monotherapy	Non-ypCR after NCRT and surgery	31/27	31/27	Pbo	38.7, survival analysis
Sakowitz et al., 2026 (31)	Retrospective national database cohort, United States	NCRT	ICI; agent not reported	Residual disease after NCRT and surgery	342/1025	NR	Ob	20.8, overall
Sugase et al., 2025 (32)	Retrospective single-center cohort, Japan	NCT	Nivolumab monotherapy	ypN-positive residual disease after NCT; pCR excluded	60/60	60/60	Ob	24.3/43.3, by arm
Verhoeven et al., 2026 (16)	Retrospective national registry cohort, Netherlands	NCRT	Nivolumab monotherapy	Residual disease after NCRT; ypT0N0 excluded	308/308	NR	Ob	24.4/31.4, by arm
Wang et al., 2026	Retrospective single-center cohort, China	NCIT	PD-1 inhibitor monotherapy	Non-pCR after NCIT and surgery	94/82	94/82	Ob	36.0, overall

Group sizes are shown as AIT/control. Chen et al. (2025) included predominantly residual disease. ypT0 was 9.5% and ypT0N0 was not reported. All other cohorts were restricted to non-pCR or residual disease.

AIT, adjuvant immunotherapy; DFS, disease-free survival; ESCC, esophageal squamous cell carcinoma; ICI, immune checkpoint inhibitor; NCIT, neoadjuvant chemoimmunotherapy; NCRT, neoadjuvant chemoradiotherapy; NCT, neoadjuvant chemotherapy; NR, not reported; Ob, observation; OS, overall survival; Pbo, placebo; PD-1, programmed cell death 1; RFS, recurrence-free survival; pCR, pathologic complete response.

Two studies were randomized trials and 11 were retrospective cohorts. Neoadjuvant treatment consisted of NCIT in six studies, NCRT in four, NCT in two, and Mixed NAT in one. NCIT and NCT studies were predominantly Asian ESCC cohorts, whereas the largest NCRT cohorts included substantial proportions of adenocarcinoma. The median follow-up ranged from 15.2 to 78.3 months (10, 28). Postoperative treatment also varied. In the source treatment cohorts reported by Geng et al., Huang et al., and Sakowitz et al., 65.6%–77.5% of patients receiving ICI-based postoperative treatment also received chemotherapy. The treatment composition and study-level assessment of immortal time bias are detailed in **Supplementary Tables S1** and **S8**. The population characteristics and risk-of-bias assessments are presented in **Supplementary Tables S2A, B**, and **S4**.

The majority of the studies restricted eligibility to patients with pathologic residual disease or provided estimates from a residual disease subgroup. The cohort in Chen et al. (24) was not formally restricted to this population, although only 16 of the 169 patients had ypT0 disease and the number of those with ypT0N0 was not reported separately.

### Main random-effects synthesis

The primary OS analysis included 12 studies and 4,225 patients (**Figure 2**). Adjuvant immunotherapy was associated with longer OS than observation or placebo (HR = 0.64, 95%CI = 0.53–0.77). Heterogeneity was moderate (*I*^2^ = 50.4%, *τ*^2^ = 0.041, *Q*-test *p* = 0.023), and the 95% prediction interval was 0.41–0.99. The DFS/RFS analysis included 11 studies and 2,363 patients. Adjuvant immunotherapy was also associated with longer DFS/RFS (HR = 0.74, 95%CI = 0.66–0.84), with an *I*^2^ of 41.4% and *τ*^2^ less than 0.001 (*Q*-test *p* = 0.073). The REML prediction interval was 0.66–0.84.

**Figure 2:**
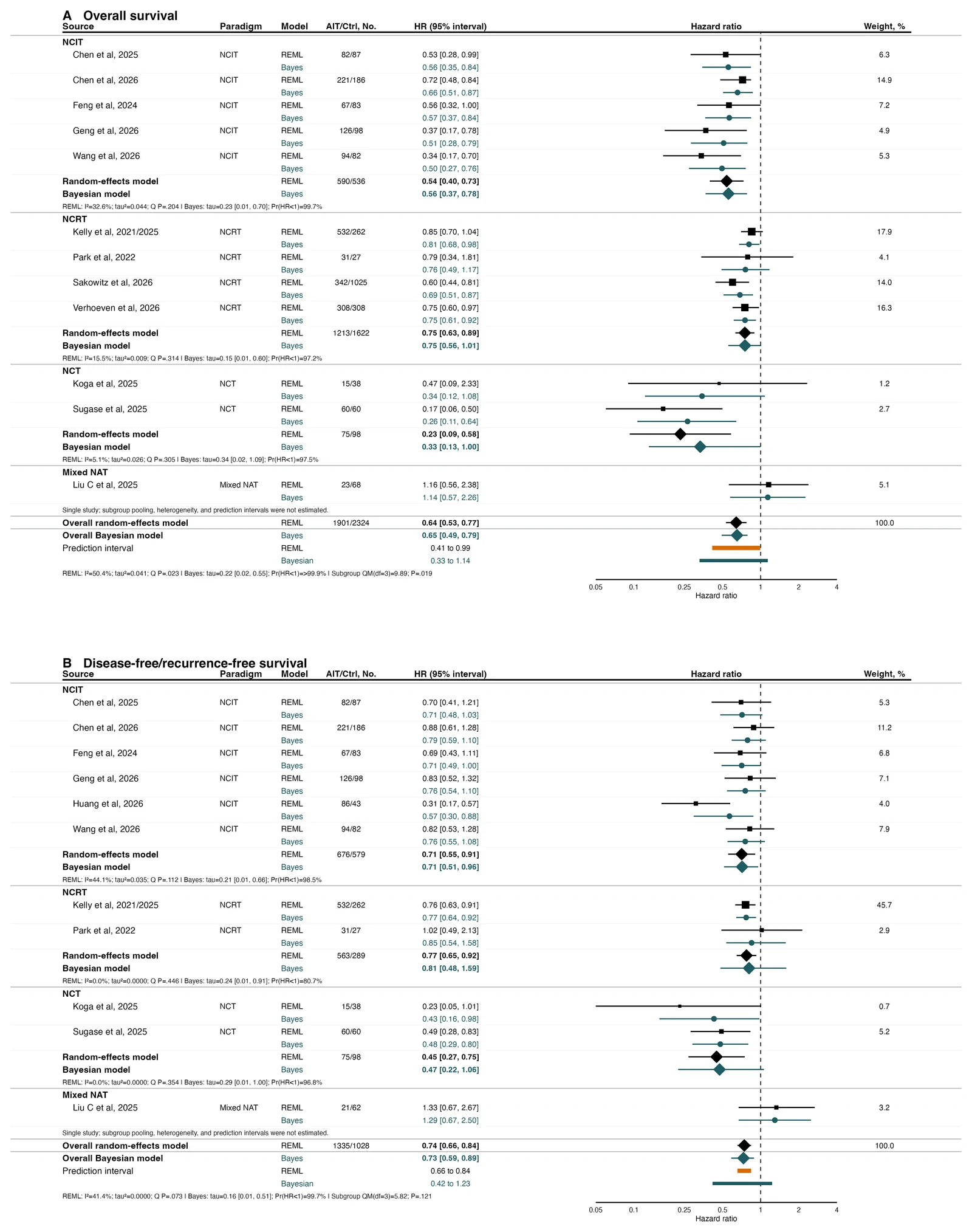
Frequentist and Bayesian meta-analysis of **(A)** overall survival and **(B)** disease-free/recurrence-free survival by neoadjuvant treatment paradigm.

The Bayesian estimates were similar for OS (HR = 0.65, 95%CrI = 0.49–0.79) and DFS/RFS (HR = 0.73, 95%CrI = 0.59–0.89). The posterior probability that the HR was below 1 exceeded 99% for both outcomes. However, the 95% Bayesian predictive intervals crossed 1 for both OS (0.33–1.14) and DFS/RFS (0.42–1.23).

Complementary analyses of reconstructed data included 10 studies with 3,340 reconstructed patient records for OS and 10 studies with 2,280 records for DFS/RFS. In the Cox models stratified by study, the estimates favored postoperative immunotherapy for OS (HR = 0.60, 95%CI = 0.52–0.69) and DFS/RFS (HR = 0.70, 95%CI = 0.61–0.79). These findings were directionally consistent with the study-level meta-analysis (**Figures 3A, B**). The study-level HR sources are summarized in **Supplementary Table S3**.

**Figure 3:**
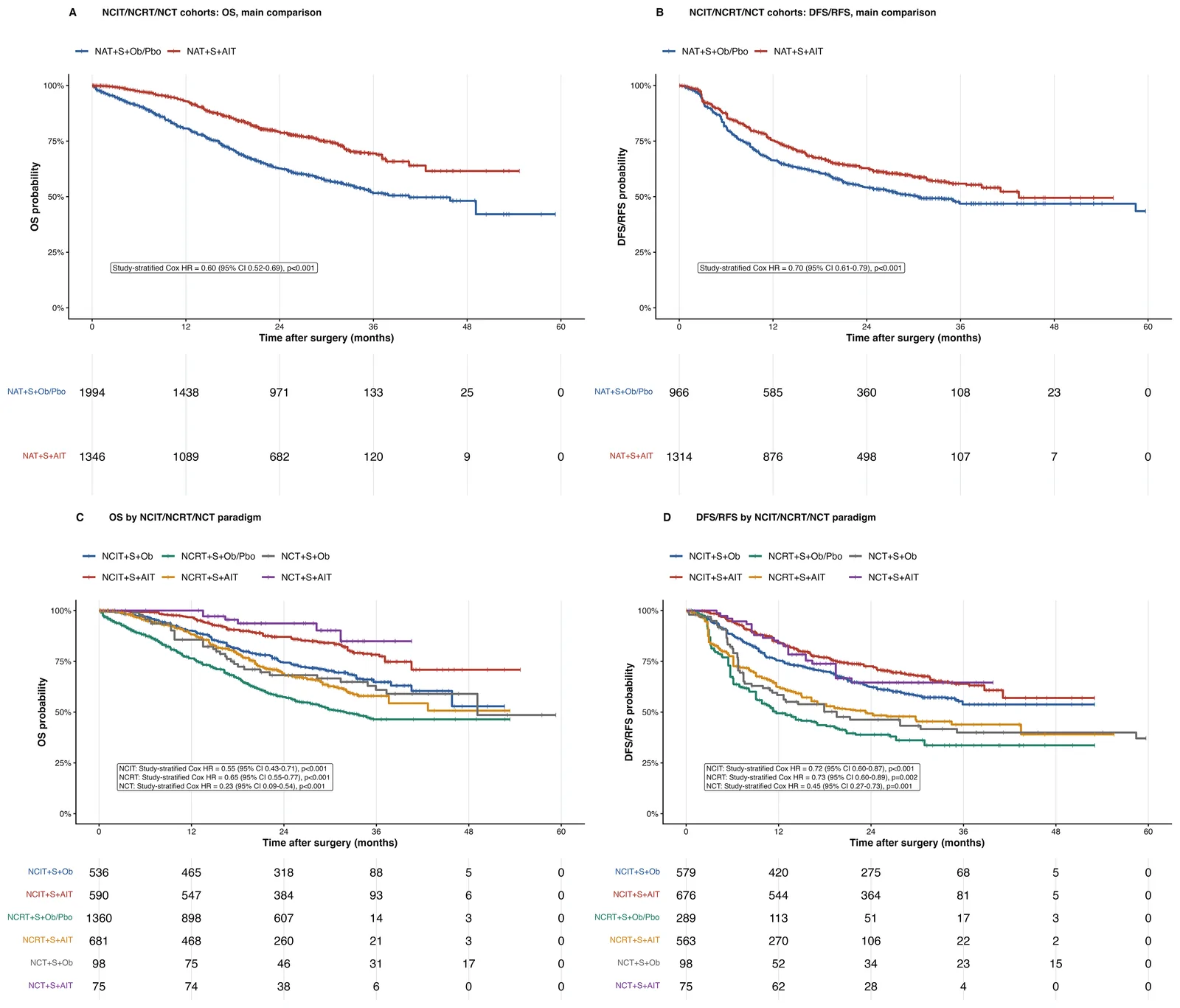
Survival curves generated from reconstructed individual participant data for the neoadjuvant chemoimmunotherapy (NCIT), neoadjuvant chemoradiotherapy (NCRT), and neoadjuvant chemotherapy (NCT) cohorts. **(A, B)** Combined overall survival (OS) and disease-free/recurrence-free survival (DFS/RFS) curves. **(C, D)** Corresponding curves by neoadjuvant treatment paradigm. Cox models combining data from multiple studies were stratified by study. Hazard ratios (HRs) compare NAT+S+AIT with NAT+S+Ob/Pbo. Tick marks indicate censoring, and numbers below each panel are patients at risk. Mixed neoadjuvant therapy *(Mixed NAT)* is not shown because the reconstructed data could not be assigned to a single neoadjuvant paradigm. AIT, adjuvant immunotherapy; CI, confidence interval; NCRT, neoadjuvant chemoradiotherapy; Ob, observation; Pbo, placebo; S, surgery.

### Paradigm-stratified random-effects synthesis

In the NCIT subgroup, the pooled HR was 0.54 for OS (95%CI = 0.40–0.73) and was 0.71 for DFS/RFS (95%CI = 0.55–0.91). All NCIT evidence came from retrospective observational studies. In the NCRT subgroup, the corresponding HRs were 0.75 for OS (95%CI = 0.63–0.89) and 0.77 for DFS/RFS (95%CI = 0.65–0.92). Randomized evidence supported the NCRT DFS/RFS estimate, whereas the pooled OS estimate also depended substantially on observational cohorts.

The exploratory NCT subgroup included two retrospective Japanese studies and 173 patients. The pooled HR was 0.23 for OS (95%CI = 0.09–0.58) and was 0.45 for DFS/RFS (95%CI = 0.27–0.75). Mixed NAT was represented by only one study and was not pooled. Its reported HRs were 1.16 for OS (95%CI = 0.56–2.38) and 1.33 for DFS/RFS (95%CI = 0.67–2.66).

The test for subgroup differences was significant for OS (Q_M = 9.89, *df* = 3, *p* = 0.019), but not for DFS/RFS (Q_M = 5.82, *df* = 3, *p* = 0.121). Because the histology, geography, study design, and neoadjuvant paradigm were closely related across studies, these results should not be interpreted as direct comparisons between neoadjuvant strategies. The Bayesian findings were generally similar, but were less precise in the smaller subgroups (**Figure 2**; **Supplementary Table S6**). The paradigm-specific reconstructed survival curves and the corresponding study-stratified Cox estimates are presented in **Figures 3C, D**.

### ESCC-predominant and residual pathology analyses

The ESCC-predominant analysis included nine studies and 1,448 patients for OS and 11 studies and 1,799 patients for DFS/RFS. Adjuvant immunotherapy remained associated with longer OS (HR = 0.55, 95%CI = 0.40–0.75) and DFS/RFS (HR = 0.70, 95%CI = 0.57–0.85) (**Figure 4**). The corresponding prediction intervals crossed 1. The tests for subgroup differences by neoadjuvant paradigm were not significant for OS (*p* = 0.079) or DFS/RFS (*p* = 0.176). The reconstructed survival curves and corresponding Cox estimates are presented in **Supplementary Figure S1**.

**Figure 4:**
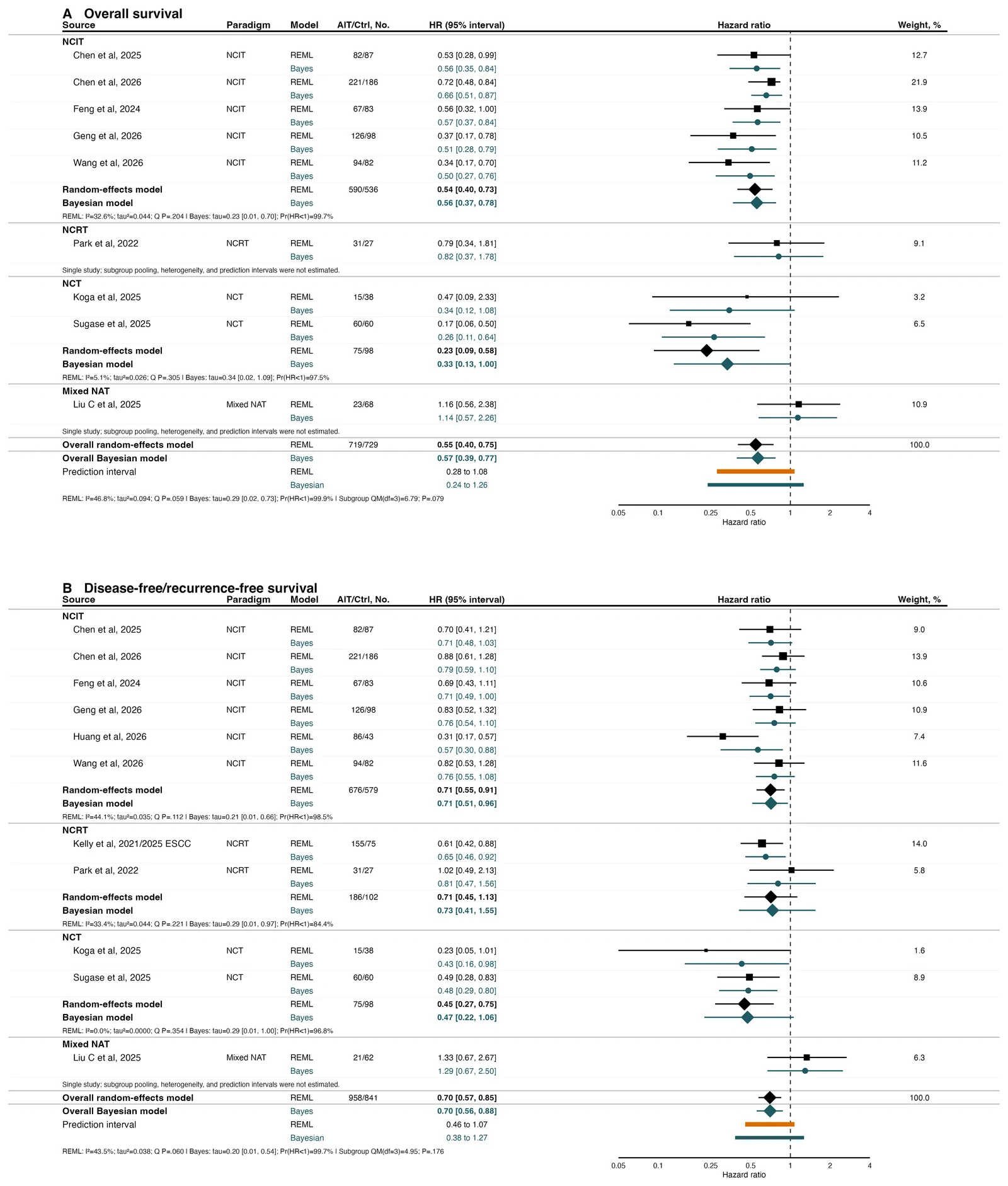
Exploratory frequentist and Bayesian meta-analysis restricted to ESCC-predominant evidence for **(A)** overall survival and **(B)** disease-free/recurrence-free survival by neoadjuvant treatment paradigm.

The residual pathology analyses were exploratory as the subgroups were small and not uniformly defined across studies (**Figure 5**). For ypT+N+ disease, the HRs were 0.46 for OS (95%CI = 0.27–0.77) and 0.91 for DFS/RFS (95%CI = 0.51–1.63). For ypT+N0-like disease, the corresponding HRs were 0.39 (95%CI = 0.15–1.03) and 0.67 (95%CI = 0.49–0.92). The estimates for ypT0N+-like disease were imprecise. Broader ypN-positive analyses remained descriptive because subgroup construction differed across studies. The reconstructed survival curves are shown in **Supplementary Figures S2** and **S3**.

**Figure 5:**
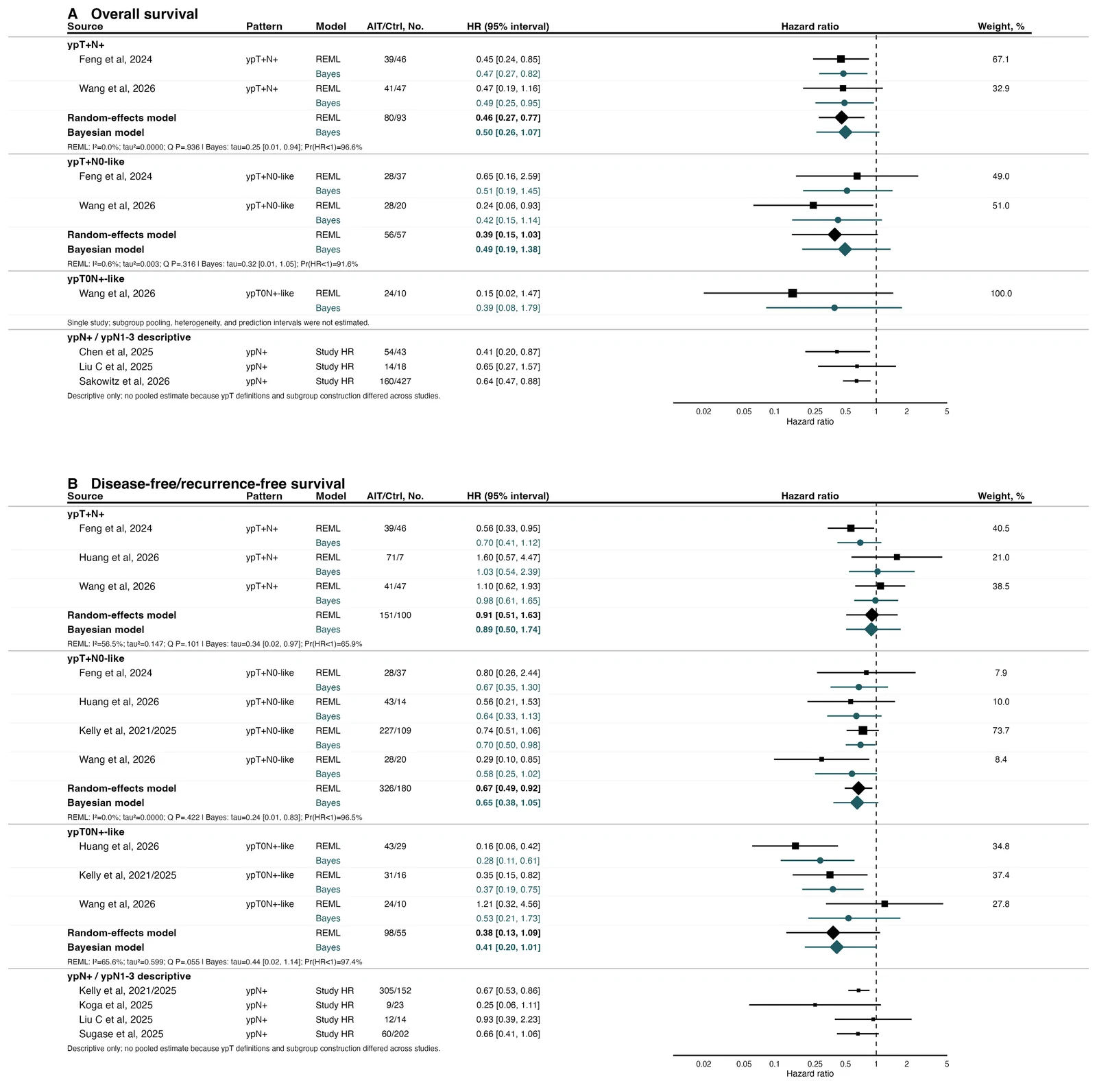
Exploratory frequentist and Bayesian meta-analysis by residual pathologic pattern for **(A)** overall survival and **(B)** disease-free/recurrence-free survival.

### Sensitivity analyses, small-study effects, and certainty of evidence

The leave-one-out analysis did not identify a study whose removal changed the direction of either outcome (**Supplementary Table S5**). The exclusion of Chen et al. (24) yielded similar estimates for OS (HR = 0.65, 95%CI = 0.53–0.79) and DFS/RFS (HR = 0.73, 95%CI = 0.60–0.88). The results also remained favorable after excluding the Kaplan–Meier-reconstructed HRs and when restricted to the reported randomized, adjusted, matched, or weighted estimates.

After excluding the three cohorts with substantial or inseparable postoperative chemotherapy use, the pooled HRs were 0.67 for OS (95%CI = 0.55–0.83) and 0.77 for DFS/RFS (95%CI = 0.67–0.87). The OS prediction interval crossed 1 (0.44–1.03).

The descriptive analysis restricted to studies with a clearly aligned time origin included three studies for OS and two for DFS/RFS. The pooled HRs were 0.81 for OS (95%CI = 0.69–0.94) and 0.77 for DFS/RFS (95%CI = 0.65–0.92). The Knapp–Hartung CI crossed 1 for DFS/RFS. A broader analysis that also included studies excluding relevant early events or using landmark methods remained favorable, although the Knapp–Hartung CI and the prediction interval crossed 1 for OS. These analyses were based on only a few studies and were considered descriptive.

Alternative heterogeneity estimators produced favorable pooled estimates, but their prediction intervals crossed 1 for both outcomes. The narrow REML prediction interval for DFS/RFS reflected an estimated *τ*^2^ close to zero and was not reproduced by the alternative estimators. The pooled estimates showed a favorable average association, but the prediction intervals crossing 1 indicated that this association may not be consistent across patient populations and treatment settings.

Egger regression was not significant for DFS/RFS (*p* = 0.262), but suggested possible small-study effects for OS (*p* = 0.003) (**Supplementary Figure S4**). After trim-and-fill, the OS estimate remained favorable (HR = 0.73, 95%CI = 0.56–0.94). GRADE certainty was low for the primary outcomes, the NCIT outcomes, and NCRT OS. Certainty was moderate for NCRT DFS/RFS and very low for NCT and Mixed NAT (**Supplementary Figure S5**; **Supplementary Table S7**).

## Discussion

In this systematic review and reconstructed IPD meta-analysis, adjuvant immunotherapy after neoadjuvant therapy and esophagectomy was associated with longer OS and DFS/RFS compared with observation or placebo. The direction of the association remained favorable across sensitivity analyses and in both the NCRT and NCIT cohorts. The Bayesian pooled estimates were similar to the frequentist estimates, although the predictive intervals crossed 1 for both outcomes, indicating uncertainty about whether the same association would be observed across different clinical settings. Interaction testing was significant for OS, but not for DFS/RFS. The OS interaction may have been influenced by the large effect estimate in the small NCT subgroup, together with the differences in the histology, study design, and patient populations across paradigms, and should not be interpreted as evidence that one neoadjuvant strategy is superior to another. The strength of evidence also differed across settings. Randomized evidence supported DFS after NCRT, whereas the evidence after NCIT was entirely observational, the evidence after NCT was limited to two small retrospective studies, and Mixed NAT was represented by only a single study. Although ESCORT-NEO/NCCES01 and HCHTOG1909 provide relevant randomized evidence for perioperative immunotherapy in resectable ESCC (8, 35), neither trial was designed to isolate the postoperative contribution of immunotherapy or compare adjuvant treatment after different neoadjuvant approaches. Whether the association with postoperative immunotherapy differs according to the preceding neoadjuvant strategy therefore remains uncertain. Our findings support further evaluation of postoperative immunotherapy as consolidation treatment for patients with pathologic residual risk, particularly in settings where randomized evidence is not yet available.

The NCRT findings are supported by the most mature evidence currently available for postoperative immunotherapy (9, 10, 16). In CheckMate 577, patients with residual disease after NCRT and surgery remained at substantial risk of recurrence and derived benefit from postoperative nivolumab, as reflected by the longer median distant metastasis-free survival with nivolumab than with placebo (27.3 *vs*. 14.6 months) (10). The favorable DFS/RFS estimate observed in the NCRT subgroup was consistent with this trial evidence. However, mature randomized OS data from CheckMate 577 did not demonstrate a statistically significant OS benefit. The favorable pooled NCRT OS estimate therefore also depended on observational cohorts, including the Dutch population-based study by Verhoeven et al. The NCRT evidence base was also histologically mixed. CheckMate 577 included both squamous cell carcinoma and adenocarcinoma or gastroesophageal junction cancer, and several large Western registry cohorts were enriched for adenocarcinoma (16, 31). Consequently, the numerically more modest OS and DFS/RFS estimates in the NCRT subgroup may reflect differences in the patient populations and treatment approaches. This interpretation is consistent with CheckMate 577, in which adjuvant nivolumab appeared to have greater benefit in ESCC than in esophageal adenocarcinoma, suggesting that the higher proportion of adenocarcinoma in the NCRT subgroup may partly explain its more modest estimates (9).

Because the histology and neoadjuvant platform were likely correlated, the analysis restricted to ESCC should be interpreted more as a sensitivity assessment than an independent test of histology-specific efficacy. After restricting the analysis to studies in which ESCC was the predominant histology, adjuvant immunotherapy remained associated with favorable OS and DFS/RFS. The overall association was therefore unlikely to be explained entirely by the NCRT cohorts that included substantial numbers of patients with adenocarcinoma. Because the majority of the NCIT studies already consisted predominantly of Asian patients with ESCC, this restriction had little effect on the NCIT estimates. In the NCRT subgroup, however, the evidence specific to ESCC remained sparse, particularly for OS. The absence of a significant OS or DFS/RFS signal in the ESCC-restricted NCRT analysis should therefore be viewed as insufficient evidence rather than as evidence of no benefit.

The NCIT findings are clinically important because randomized evidence has not yet clarified whether immunotherapy should be continued after surgery in patients already exposed to preoperative PD-1 or PD-L1 blockade. Both the OS and DFS/RFS estimates favored postoperative immunotherapy after NCIT. However, all evidence in this setting came from observational studies. Treatment allocation may have been influenced by postoperative recovery, residual disease burden, physician judgment, and institutional practice. Patients also had to survive and recover sufficiently to begin postoperative immunotherapy, making these comparisons vulnerable to immortal time bias. This bias is not necessarily removed by matching, weighting, or conventional multivariable adjustment. Across the available evidence, the estimates remained favorable when the analyses were restricted to studies with an aligned time origin or an explicit approach to reduce this bias, although only a small number of studies met these criteria (**Supplementary Tables S5**, **S8**).

The NCIT estimates suggest that prior exposure to an ICI does not necessarily eliminate the potential value of postoperative treatment, but they do not support routine continuation for every patient. Patients who achieve pCR generally have favorable outcomes, which may leave less room for additional postoperative treatment. In the weighted analysis by Geng et al., adjuvant immunotherapy was not associated with longer OS, DFS, or cancer-specific survival among patients who achieved pCR after NCIT (26). Liu et al. similarly found no association between postoperative adjuvant therapy and DFS or OS in the pCR subgroup, although the postoperative treatments included chemotherapy, radiotherapy, and immunochemotherapy and did not isolate the effect of ICI (34). The additional value of postoperative ICI after pCR therefore remains uncertain. In contrast, the association appeared more evident among patients with residual disease, particularly those with residual nodal involvement (23, 25, 26, 33). Pathologic residual risk may therefore help identify patients for whom further immunotherapy warrants consideration after NCIT.

The exploratory residual pathology analyses, which included evidence from different neoadjuvant settings, examined the association with postoperative immunotherapy within several patterns of residual disease. In the ypT+N+ subgroup, postoperative immunotherapy was associated with longer OS, whereas the DFS/RFS estimate remained uncertain. For ypT0N+-like disease, both the OS and DFS/RFS estimates favored postoperative immunotherapy, but neither estimate was precise. For ypT+N0-like disease, the DFS/RFS estimate was favorable, whereas the OS estimate remained imprecise. The ypT+N0-like DFS/RFS analysis included the CheckMate 577 ypN0 subgroup (9). Because CheckMate 577 enrolled only patients with residual disease, those with ypN0 necessarily had residual disease in the primary tumor and were therefore considered ypT+N0-like in the present analysis. However, the trial reported ypN0 rather than a specifically defined ypT+N0 subgroup. The broader ypN+/ypN1–3 analysis remained descriptive because the ypT composition and subgroup construction differed across studies. These findings remain exploratory, but suggest that the residual nodal status and combined ypT+N+ disease may be clinically relevant when postoperative immunotherapy is considered, particularly after NCIT, where the evidence remains limited.

The possible importance of residual nodal status is also consistent with previous studies. Raja et al. suggested that patients with ypN2–3 disease may derive greater benefit from postoperative therapy (35). In the multicenter NCIT cohort, Chen et al. suggested that patients with ypN0 disease may have limited need for additional immunotherapy, whereas ypN2–3 disease may reflect resistant biology for which postoperative immunotherapy alone is inadequate. The apparent benefit was greatest in ypN1 disease (23). The relationship between residual disease burden and benefit from postoperative immunotherapy may therefore not be simply dose-dependent. A clinically relevant group may instead be patients with residual disease, especially residual nodal disease, who remain at high risk after surgery but may still respond to systemic consolidation (36, 37). Further studies will need to evaluate the ypN strata, nodal downstaging, tumor regression, circulating tumor DNA, and minimal residual disease biomarkers to refine postoperative treatment selection (36–39).

In Japan, NCT, particularly DCF (docetaxel, cisplatin, 5-fluorouracil), is an established preoperative treatment for resectable locally advanced ESCC following the results of JCOG1109 (5). Koga and Sugase examined adjuvant nivolumab after NCT, a setting not directly evaluated in CheckMate 577 (9, 28, 32). Sugase et al. reported associations with longer DFS and OS after propensity score matching. In the exploratory analyses, the DFS association appeared more pronounced in patients with ypT3, ypN2, or ypStage III disease (32). Koga et al. included several chemotherapy regimens, most commonly DCF, and reported a favorable DFS pattern with adjuvant nivolumab, particularly among patients with residual nodal disease (28). However, the difference was no longer statistically significant in a 6-month landmark analysis undertaken to reduce immortal time bias. The selected analytic cohorts contributed 173 patients to the NCT subgroup, and neither study provided a randomized comparison. These findings suggest that adjuvant nivolumab after NCT may be relevant for patients with residual disease, particularly residual nodal disease. However, the evidence remains preliminary.

Mixed NAT was represented by a single retrospective study by Liu et al. (29). The majority of patients received chemoradiotherapy before surgery, while others received chemotherapy alone. The postoperative intervention was ICI monotherapy, although the specific agent was not reported. Because the cohort combined different preoperative treatments and produced imprecise estimates, it could not clarify the association with postoperative immunotherapy after any individual neoadjuvant approach.

The variation was not limited to preoperative treatment. The postoperative regimens also differed across a small number of studies. Majority of the included comparisons involved ICI monotherapy or a separately defined ICI treatment arm. Substantial concomitant postoperative chemotherapy was present in a minority of comparisons, affecting two of 12 studies contributing to OS and two of 11 studies contributing to DFS/RFS. These studies accounted for 18.9% and 11.1% of the respective meta-analytic weights. In the cohorts reported by Geng, Huang, and Sakowitz, 65.6%, 72.8%, and 77.5% of patients receiving ICI-based postoperative treatment also received chemotherapy, respectively (26, 27, 31). Because chemotherapy was frequently co-administered in these three cohorts, the corresponding study-level estimates may reflect the combined effects of ICI and chemotherapy rather than the effect of ICI monotherapy alone. After excluding these cohorts, the pooled average associations remained favorable for OS and DFS/RFS, although the estimates were modestly attenuated (**Supplementary Table S5**). The 95% prediction interval for OS was 0.44–1.03 in this sensitivity analysis, indicating that the favorable average OS association may not be reproduced in every new clinical setting.

Two practical issues also deserve consideration in the postoperative setting. Adjuvant treatment is intended to control occult micrometastatic disease after surgery, which might favor early treatment initiation. In CheckMate 577, however, the subgroup estimate appeared more favorable among patients who began nivolumab at least 10 weeks after surgery than among those who started earlier (9). This observation should be interpreted with caution because the treatment timing was not randomized and may have reflected postoperative recovery, complications, and patient selection rather than a true advantage of delayed initiation. The biomarker status may also change during neoadjuvant treatment. Park et al. reported the conversion of PD-L1 expression after NCRT, including changes between positive and negative status (30). Pretreatment samples may therefore not fully represent the residual tumor targeted by postoperative immunotherapy. These observations support considering postoperative condition, residual pathology, and updated biomarker information rather than relying on a single pretreatment factor.

Limitations should be acknowledged. Of the 13 studies included in the quantitative synthesis, 11 studies were retrospective. Adjusted, matched, or weighted estimates were prioritized when available, and the direction of the findings remained consistent across multiple sensitivity analyses. Nevertheless, treatment selection bias and unmeasured confounding could not be eliminated. Immortal time bias also remained possible because a few studies aligned the postoperative time origin or used landmark or time-dependent methods, although the analyses addressing treatment timing were directionally consistent. In a number of studies, patients receiving postoperative immunotherapy were treated more recently than the control patients; therefore, improvements in surgery, perioperative care, surveillance, or treatment after recurrence may have contributed to the observed survival differences. Substantial concomitant chemotherapy was present in a small minority of postoperative ICI comparisons, affecting two of 12 studies contributing to OS and two of 11 studies contributing to DFS/RFS. The exclusion of these studies did not materially alter the favorable average associations. Clinical heterogeneity remained across histology, geography, treatment regimens, outcome definitions, and follow-up maturity. Follow-up ranged from 15.2 to 78.3 months, and shorter follow-up may have captured recurrence more reliably than mature OS. One cohort was not formally restricted to patients with residual disease, but was predominantly composed of such patients. Only 9.5% had ypT0 disease, and ypT0N0 was not reported separately, meaning that the actual pCR proportion could not have exceeded 9.5%. The exclusion of this cohort did not materially change the pooled estimates. A small number of study-level HRs were estimated from published Kaplan–Meier curves, while the aggregated survival curves were based on reconstructed rather than original IPD. The residual pathology analyses remained exploratory due to the small sample sizes and inconsistent subgroup definitions. Predictive intervals from several frequentist and Bayesian models crossed 1, indicating that the favorable average association may not extend to every new clinical setting. Funnel plot asymmetry suggested possible small-study effects for OS. The safety, treatment completion, quality of life, and nutritional recovery could not be synthesized. These limitations contributed to the low certainty of evidence for the primary outcomes.

Taken together, the findings indicate a favorable average association between postoperative immunotherapy and survival, but the strength of evidence differs across neoadjuvant settings. After NCRT, randomized evidence from CheckMate 577 supports a DFS benefit from adjuvant nivolumab in patients with residual disease, although mature randomized data have not demonstrated a statistically significant OS benefit (9). The favorable pooled NCRT OS estimate also depended on observational cohorts. After NCIT, all available evidence was observational and does not establish routine continuation of immunotherapy for every patient. The evidence after NCT remains preliminary, while Mixed NAT was represented by a single heterogeneous cohort. The residual pathology findings may help frame future patient selection, but remain exploratory. Randomized studies after preoperative PD-1 or PD-L1 blockade will be needed to account for postoperative recovery and treatment timing and to evaluate the relevance of residual pathology and evolving biomarkers (9, 30). The central clinical question may be not whether immunotherapy should be continued routinely after surgery, but which patients remain at sufficient risk to justify further treatment.

## Conclusions

Among the cohorts composed predominantly of patients with residual disease after neoadjuvant therapy and esophagectomy, postoperative immunotherapy was associated with longer OS and DFS/RFS than observation or placebo. The association remained directionally favorable across sensitivity analyses. However, the overall certainty of evidence was low, and the prediction intervals crossed 1 in several frequentist and Bayesian models, indicating uncertainty about whether similar associations would be observed across different clinical settings. After NCRT, randomized evidence supports a DFS benefit from adjuvant immunotherapy. The pooled NCRT OS estimate was also favorable; however, a statistically significant OS benefit has not been demonstrated in randomized trials, and the pooled estimate was partly supported by observational cohorts. The pooled estimates after NCIT and NCT were favorable, but the NCIT evidence was entirely observational and the NCT evidence was limited to two small retrospective studies. These paradigm-specific estimates should not be interpreted as direct comparisons because the histology, geography, and patient populations differed across neoadjuvant strategies. Mixed NAT was represented by a single study with imprecise estimates. The residual pathology findings were exploratory and require prospective validation. Further randomized studies are needed to define the role of postoperative immunotherapy after NCIT or NCT and to determine whether pathologic residual features can inform patient selection.

## Data Availability

The original contributions presented in the study are included in the article/**Supplementary Material**. Further inquiries can be directed to the corresponding author.

## Supplementary material

Supplementary Figure 1 Survival curves generated from reconstructed individual participant data for ESCC-predominant NCIT, NCRT, and NCT cohorts. Panels **(A, B)** show the combined OS and DFS/RFS curves. Panels **(C, D)** show the corresponding curves by neoadjuvant treatment paradigm. Cox models combining data from multiple studies were stratified by study. A conventional Cox model was used for the single-study NCRT OS analysis. HRs compare NAT+S+AIT with NAT+S+Ob/Pbo. Tick marks indicate censoring, and numbers below each panel are patients at risk. Mixed NAT was not shown because the reconstructed data could not be assigned to a single neoadjuvant paradigm. AIT indicates adjuvant immunotherapy; CI, confidence interval; DFS, disease-free survival; ESCC, esophageal squamous cell carcinoma; HR, hazard ratio; Mixed NAT, mixed neoadjuvant therapy; NAT, neoadjuvant therapy; NCIT, neoadjuvant chemoimmunotherapy; NCRT, neoadjuvant chemoradiotherapy; NCT, neoadjuvant chemotherapy; Ob, observation; OS, overall survival; Pbo, placebo; RFS, recurrence-free survival; S, surgery.

Supplementary Figure 2 Overall survival curves generated from reconstructed individual participant data by residual pathologic pattern. Cox models were stratified by study for panels combining multiple studies, and conventional Cox models were used for single-study panels. HRs compare NAT+S+AIT with NAT+S+Ob/Pbo. Tick marks indicate censoring, and numbers below each panel are patients at risk. AIT indicates adjuvant immunotherapy; CI, confidence interval; HR, hazard ratio; NAT, neoadjuvant therapy; Ob, observation; OS, overall survival; Pbo, placebo; S, surgery; ypN, post-neoadjuvant pathologic nodal category; ypT, post-neoadjuvant pathologic primary-tumor category.

Supplementary Figure 3 Disease-free or recurrence-free survival curves generated from reconstructed individual participant data by residual pathologic pattern. Cox models were stratified by study for panels combining multiple studies. HRs compare NAT+S+AIT with NAT+S+Ob/Pbo. Tick marks indicate censoring, and numbers below each panel are patients at risk. AIT indicates adjuvant immunotherapy; CI, confidence interval; DFS, disease-free survival; HR, hazard ratio; NAT, neoadjuvant therapy; Ob, observation; Pbo, placebo; RFS, recurrence-free survival; S, surgery; ypN, post-neoadjuvant pathologic nodal category; ypT, post-neoadjuvant pathologic primary-tumor category.

Supplementary Figure 4 Funnel plots for OS and DFS/RFS, including main and exploratory paradigm-stratified displays.

Supplementary Figure 5 GRADE evidence profile for disease-free/recurrence-free survival and overall survival.
