# Supplementary material for: Adjuvant immunotherapy versus observation after neoadjuvant therapy and esophagectomy for esophageal cancer with residual pathologic disease: a systematic review and reconstructed individual participant data meta-analysis
Source: Front Immunol. 2026 Sep 24;17:1944032. doi: 10.3389/fimmu.2026.1944032 (PMC13646074; doi:10.3389/fimmu.2026.1944032)
Supplement: Supplementary file 1 [file Table1.docx]

**Table S1. Neoadjuvant, surgical, and postoperative treatment regimens**

| **Study** | **Paradigm** | **Neoadjuvant treatment** | **Surgery** | **Postoperative treatment in intervention arm** | **Comparator** | **Planned AIT duration** |
| --- | --- | --- | --- | --- | --- | --- |
| Chen et al, 2025 | NCIT | PD-1 inhibitor plus paclitaxel-platinum chemotherapy, 2-4 cycles | Esophagectomy with curative intent | PD-1 inhibitor | Observation/no adjuvant immunotherapy | NR |
| Chen et al, 2026 | NCIT | Taxane-platinum chemotherapy plus PD-1 inhibitor, 2-4 cycles | R0 esophagectomy | PD-1 inhibitor | Observation/no adjuvant immunotherapy | 1 year |
| Feng et al, 2024 | NCIT | PD-1 inhibitor plus chemotherapy; multiple PD-1 agents | Radical esophagectomy | PD-1 inhibitor | Observation/no adjuvant immunotherapy | 1-2 years when used |
| Geng et al, 2026 | NCIT | Taxane-platinum chemotherapy plus PD-1/PD-L1 inhibitor | Esophagectomy | ICI monotherapy or ICI plus chemotherapy | Observation/no adjuvant immunotherapy | NR |
| Huang et al, 2026 | NCIT | Taxane-platinum chemotherapy plus PD-1/PD-L1 inhibitor | Esophagectomy | ICI monotherapy or ICI plus chemotherapy | Observation/no adjuvant therapy | 1 year |
| Kelly et al, 2021/2025 | NCRT | Neoadjuvant chemoradiotherapy, commonly carboplatin-paclitaxel-based | R0 resection | Nivolumab monotherapy | Placebo | Up to 1 year |
| Koga et al, 2025 | NCT | Neoadjuvant chemotherapy, mainly DCF/FP/FOLFOX | Esophagectomy | Nivolumab monotherapy | Observation/no adjuvant nivolumab | Up to 1 year |
| Liu C et al, 2025 | Mixed NAT | Mixed neoadjuvant therapy; most patients received chemoradiotherapy, some chemotherapy alone | Esophagectomy | ICI monotherapy; agent not reported | Observation/no adjuvant therapy | NR |
| Park et al, 2022 | NCRT | 5-FU plus cisplatin with 44 Gy radiotherapy | Curative surgery | Durvalumab monotherapy | Placebo | 1 year |
| Sakowitz et al, 2026 | NCRT | Neoadjuvant chemoradiotherapy in NCDB | Esophagectomy with complete resection | ICI; agent not reported | Observation/no adjuvant immunotherapy | NR |
| Sugase et al, 2025 | NCT | neoDCF: docetaxel, cisplatin, and 5-FU | Esophagectomy | Nivolumab monotherapy | Observation/no adjuvant nivolumab | Up to 1 year |
| Verhoeven et al, 2026 | NCRT | CROSS-type carboplatin-paclitaxel plus 41.4 Gy radiotherapy | Esophagectomy | Nivolumab monotherapy | Observation/no adjuvant nivolumab | 1 year when completed |
| Wang et al, 2026 | NCIT | Paclitaxel-platinum chemotherapy plus PD-1 inhibitor | Esophagectomy | PD-1 inhibitor monotherapy | Observation/no adjuvant immunotherapy | NR |

Abbreviations: ACT, adjuvant chemotherapy; AICT, adjuvant immunochemotherapy; AIT, adjuvant immunotherapy; ART, adjuvant radiotherapy; DCF, docetaxel, cisplatin, and fluorouracil; FOLFOX, fluorouracil, leucovorin, and oxaliplatin; ICI, immune checkpoint inhibitor; NCDB, National Cancer Database; NCIT, neoadjuvant chemoimmunotherapy; NCRT, neoadjuvant chemoradiotherapy; NCT, neoadjuvant chemotherapy; NR, not reported; PAIC, postoperative adjuvant immunochemotherapy; PD-1, programmed cell death 1; PD-L1, programmed death-ligand 1. For Geng, Huang, and Sakowitz, chemotherapy proportions refer to the source treatment cohort because the exact proportion in the meta-analysis subset was not reported.
