# Supplementary material for: Adjuvant immunotherapy versus observation after neoadjuvant therapy and esophagectomy for esophageal cancer with residual pathologic disease: a systematic review and reconstructed individual participant data meta-analysis
Source: Front Immunol. 2026 Sep 24;17:1944032. doi: 10.3389/fimmu.2026.1944032 (PMC13646074; doi:10.3389/fimmu.2026.1944032)
Supplement: Supplementary file 2 [file Table2.docx]

**Supplementary Table S2. Characteristics of the included study populations: Part A, demographic and clinical characteristics**

| **Study** | **Arm** | **Baseline N** | **Baseline population** | **Meta-analysis population** | **Age** | **Male sex** | **ECOG/PS** | **BMI** | **Smoking** | **Alcohol** | **Comorbidity** |
| --- | --- | --- | --- | --- | --- | --- | --- | --- | --- | --- | --- |
| Chen et al, 2025 | AIT | 82 | Predominantly residual-disease post-NCIT R0 ESCC cohort; ypT0 occurred in 16/169 patients (9.5%), while strict ypT0N0 pCR was not reported | OS 82; DFS/RFS 82 | Mean 60.24 ± 5.80 (pooled from TRG strata) | 61/82 (74.4%) | NR | Mean 21.77 ± 3.50 (pooled from TRG strata) | NR | NR | NR |
| Chen et al, 2025 | Control | 87 | Predominantly residual-disease post-NCIT R0 ESCC cohort; ypT0 occurred in 16/169 patients (9.5%), while strict ypT0N0 pCR was not reported | OS 87; DFS/RFS 87 | Mean 63.12 ± 5.97 (pooled from TRG strata) | 69/87 (79.3%) | NR | Mean 21.84 ± 2.71 (pooled from TRG strata) | NR | NR | NR |
| Chen et al, 2026 | AIT | 262 | Matched overall post-NCIT cohort | OS 221; DFS/RFS 221 | <60: 74/262 (28.2%); ≥60: 188/262 (71.8%) | 209/262 (79.8%) | ECOG 0: 126/262 (48.1%); ECOG 1: 136/262 (51.9%) | <18: 12/262 (4.6%); 18-24: 147/262 (56.1%); >24: 103/262 (39.3%) | 171/262 (65.3%) | 187/262 (71.4%) | NR |
| Chen et al, 2026 | Control | 262 | Matched overall post-NCIT cohort | OS 186; DFS/RFS 186 | <60: 78/262 (29.8%); ≥60: 184/262 (70.2%) | 202/262 (77.1%) | ECOG 0: 121/262 (46.2%); ECOG 1: 141/262 (53.8%) | <18: 13/262 (5.0%); 18-24: 150/262 (57.2%); >24: 99/262 (37.8%) | 169/262 (64.5%) | 183/262 (69.9%) | NR |
| Feng et al, 2024 | AIT | 39 | Reported ypT+N+ subgroup | OS 67; DFS/RFS 67 | Median 64 (IQR 57-69) | 89.7% | NR | Median 21.8 (IQR 20.2-23.6) vs 21.6 (IQR 19.6-22.6) | NR | NR | NR |
| Feng et al, 2024 | Control | 46 | Reported ypT+N+ subgroup | OS 83; DFS/RFS 83 | Median 63 (IQR 57-68) | 82.6% | NR | Median 21.8 (IQR 20.2-23.6) vs 21.6 (IQR 19.6-22.6) | NR | NR | NR |
| Geng et al, 2026 | AIT | 126 | non-pCR subgroup (after sIPTW) | OS 126; DFS/RFS 126 | ≥65yr: 42.0% (AIT) vs 41.1% (observation/no AIT) | 79.7% (AIT) vs 79.7% (observation/no AIT) after sIPTW | NR | NR | Smoking: 56.1% vs 55.8% (after sIPTW) | Drinking: 43.3% vs 43.1% (after sIPTW) | NR |
| Geng et al, 2026 | Control | 98 | non-pCR subgroup (after sIPTW) | OS 98; DFS/RFS 98 | ≥65yr: 42.0% (AIT) vs 41.1% (observation/no AIT); age dichotomized only | 79.7% (AIT) vs 79.7% (observation/no AIT) after sIPTW | NR | NR | Smoking: 56.1% vs 55.8% (after sIPTW) | Drinking: 43.3% vs 43.1% (after sIPTW) | NR |
| Huang et al, 2026 | AIT | 283 | Overall post-NCIT cohort | DFS/RFS 86 | Mean 67.2 ± 7.2 (overall cohort) | 196/283 (69.3%; overall cohort) | NR | Mean 21.1 ± 3.3 (overall cohort) | NR | NR | NR |
| Huang et al, 2026 | Control | 283 | Overall post-NCIT cohort | DFS/RFS 43 | Mean 67.2 ± 7.2 (overall cohort) | 196/283 (69.3%; overall cohort) | NR | Mean 21.1 ± 3.3 (overall cohort) | NR | NR | NR |
| Kelly et al, 2021/2025 | AIT | 532 | ITT: non-pCR post-NCRT+R0 RCT | OS 532; DFS/RFS 532 | Median 62 (range 26-82) | 84.0% | ECOG 0: 58% vs 60%; ECOG 1: 42% vs 40% | NR | NR | NR | NR |
| Kelly et al, 2021/2025 | Control | 262 | ITT: non-pCR post-NCRT+R0 RCT | OS 262; DFS/RFS 262 | Median 61 (range 26-86) | 85.0% | ECOG 0: 58% vs 60%; ECOG 1: 42% vs 40% | NR | NR | NR | NR |
| Koga et al, 2025 | AIT | 15 | Non-pCR population after neoadjuvant chemotherapy and esophagectomy | OS 15; DFS/RFS 15 | Median 68 (IQR 62-72) | 86.7% | ECOG 0: 93.3% vs 97.4% | NR | NR | NR | NR |
| Koga et al, 2025 | Control | 38 | Non-pCR population after neoadjuvant chemotherapy and esophagectomy | OS 38; DFS/RFS 38 | Median 69 (IQR 63.8-72.3) | 78.9% | ECOG 0: 93.3% vs 97.4% | NR | NR | NR | NR |

**Supplementary Table S2. Characteristics of the included study populations: Part A (continued)**

| **Study** | **Arm** | **Baseline N** | **Baseline population** | **Meta-analysis population** | **Age** | **Male sex** | **ECOG/PS** | **BMI** | **Smoking** | **Alcohol** | **Comorbidity** |
| --- | --- | --- | --- | --- | --- | --- | --- | --- | --- | --- | --- |
| Liu C et al, 2025 | AIT | 23 | non-pCR cohort (three-group comparison) | OS 23; DFS/RFS 21 | Mean 60.2±8.5 | 100.0% | ECOG 0: 91.3% vs 86.8% (no adj) | NR | NR | NR | NR |
| Liu C et al, 2025 | Control | 68 | non-pCR cohort (three-group comparison) | OS 68; DFS/RFS 62 | Mean 60.4±8.2 | 79.4% | ECOG 0: 91.3% vs 86.8% (no adj) | NR | NR | NR | NR |
| Park et al, 2022 | AIT | 45 | ITT: all post-NCRT+surgery (Phase II RCT) | OS 31; DFS/RFS 31 | Median 62.5 (IQR 57-68) | 88.9% | ECOG 0: 68.9% vs 73.2%; ECOG 1: 31.1% vs 26.8% | Mean 23.8 vs 23.5 | Smoking ever: 77.8% vs 73.2% | Alcohol ever: 77.8% vs 70.7% | NR |
| Park et al, 2022 | Control | 41 | ITT: all post-NCRT+surgery (Phase II RCT) | OS 27; DFS/RFS 27 | Median 62 (IQR 58-67) | 85.4% | ECOG 0: 68.9% vs 73.2%; ECOG 1: 31.1% vs 26.8% | Mean 23.8 vs 23.5 | Smoking ever: 77.8% vs 73.2% | Alcohol ever: 77.8% vs 70.7% | NR |
| Sakowitz et al, 2026 | AIT | 342 | non-pCR (ypT+ or ypN+) NCDB cohort | OS 342 | Median 64 (IQR 57-69) | 83.0% | NR | NR | NR | NR | CDI 0-1: 88% vs 87%; CDI 2-3: 12% vs 13% |
| Sakowitz et al, 2026 | Control | 1025 | non-pCR (ypT+ or ypN+) NCDB cohort | OS 1025 | Median 65 (IQR 59-71) | 84.0% | NR | NR | NR | NR | CDI 0-1: 88% vs 87%; CDI 2-3: 12% vs 13% |
| Sugase et al, 2025 | AIT | 60 | ypN-positive residual disease after neoadjuvant chemotherapy and surgery; pCR excluded by definition | OS 60; DFS/RFS 60 | Median 65 (IQR 60-70) | 70.0% | NR | NR | NR | NR | NR |
| Sugase et al, 2025 | Control | 60 | ypN-positive residual disease after neoadjuvant chemotherapy and surgery; pCR excluded by definition | OS 60; DFS/RFS 60 | Median 66.5 (IQR 59-72) | 68.0% | NR | NR | NR | NR | NR |
| Verhoeven et al, 2026 | AIT | 308 | Residual disease after NCRT and surgery; complete pathologic response (ypT0N0) excluded | OS 308 | Median 65 (IQR 59-70) | 82.8% | NR | NR | NR | NR | CCI ≥1: 41.9% vs 38.3% |
| Verhoeven et al, 2026 | Control | 308 | Residual disease after NCRT and surgery; complete pathologic response (ypT0N0) excluded | OS 308 | Median 66 (IQR 59-71) | 81.8% | NR | NR | NR | NR | CCI ≥1: 41.9% vs 38.3% |
| Wang et al, 2026 | AIT | NR | NPCR classification cohort | OS 94; DFS/RFS 94 | Median 63 (range 39-77) | 86.4% (overall 243) | NR | NR | Smoking ever: 59.7% (overall) | Drinking ever: 63.4% (overall) | NR |
| Wang et al, 2026 | Control | NR | NPCR classification cohort | OS 82; DFS/RFS 82 | Median 63 (range 39-77) | 86.4% (overall 243) | NR | NR | Smoking ever: 59.7% (overall) | Drinking ever: 63.4% (overall) | NR |

*Baseline N and characteristics refer to the population identified in the Baseline population column; endpoint-specific meta-analysis sample sizes are shown separately.*

*Abbreviations: AIT, adjuvant immunotherapy; BMI, body mass index; ECOG, Eastern Cooperative Oncology Group; NR, not reported; PS, performance status.*

**Supplementary Table S2. Characteristics of the included study populations: Part B, tumor and pathologic characteristics**

| **Study** | **Arm** | **Baseline N** | **Baseline population** | **Meta-analysis population** | **Histology** | **Tumor location** | **Clinical stage** | **ypT stage** | **ypN stage** | **Pathologic stage** | **Residual disease/pCR** |
| --- | --- | --- | --- | --- | --- | --- | --- | --- | --- | --- | --- |
| Chen et al, 2025 | AIT | 82 | Predominantly residual-disease post-NCIT R0 ESCC cohort; ypT0 occurred in 16/169 patients (9.5%), while strict ypT0N0 pCR was not reported | OS 82; DFS/RFS 82 | ESCC 100%; adenocarcinoma 0% | Upper 13/82 (15.9%); middle 42/82 (51.2%); lower 27/82 (32.9%) | cT2 21/82 (25.6%); cT3 44/82 (53.7%); cT4 17/82 (20.7%) | ypT0 9/82 (11.0%); ypT1 27/82 (32.9%); ypT2 23/82 (28.0%); ypT3 23/82 (28.0%); ypT4 0/82 (0%) | ypN0 28/82 (34.1%); ypN1 40/82 (48.8%); ypN2 13/82 (15.9%); ypN3 1/82 (1.2%) | NR | ypT0 occurred in 16/169 patients (9.5%) overall; strict ypT0N0 pCR was not reported, so the pCR proportion was no greater than 9.5% |
| Chen et al, 2025 | Control | 87 | Predominantly residual-disease post-NCIT R0 ESCC cohort; ypT0 occurred in 16/169 patients (9.5%), while strict ypT0N0 pCR was not reported | OS 87; DFS/RFS 87 | ESCC 100%; adenocarcinoma 0% | Upper 8/87 (9.2%); middle 43/87 (49.4%); lower 36/87 (41.4%) | cT2 33/87 (37.9%); cT3 37/87 (42.5%); cT4 17/87 (19.5%) | ypT0 7/87 (8.0%); ypT1 40/87 (46.0%); ypT2 19/87 (21.8%); ypT3 20/87 (23.0%); ypT4 1/87 (1.1%) | ypN0 44/87 (50.6%); ypN1 26/87 (29.9%); ypN2 14/87 (16.1%); ypN3 3/87 (3.4%) | NR | ypT0 occurred in 16/169 patients (9.5%) overall; strict ypT0N0 pCR was not reported, so the pCR proportion was no greater than 9.5% |
| Chen et al, 2026 | AIT | 262 | Matched overall post-NCIT cohort | OS 221; DFS/RFS 221 | ESCC 100%; adenocarcinoma 0% | Proximal 32/262 (12.2%); middle 153/262 (58.4%); distal 77/262 (29.4%) | cT1 5/262 (1.9%); cT2 22/262 (8.4%); cT3 219/262 (83.6%); cT4a 16/262 (6.1%); cN+ 221/262 (84.4%) | Detailed ypT distribution NR in main text | ypN+ 50.8% in matched overall cohort | AJCC pStage III/IV 51.9% in matched overall cohort | Non-pCR 221/262 (84.4%); the meta-analysis used the non-pCR subgroup |
| Chen et al, 2026 | Control | 262 | Matched overall post-NCIT cohort | OS 186; DFS/RFS 186 | ESCC 100%; adenocarcinoma 0% | Proximal 30/262 (11.4%); middle 153/262 (58.4%); distal 79/262 (30.2%) | cT1 2/262 (0.8%); cT2 20/262 (7.6%); cT3 216/262 (82.4%); cT4a 24/262 (9.2%); cN+ 224/262 (85.5%) | Detailed ypT distribution NR in main text | ypN+ 38.9% in matched overall cohort | AJCC pStage III/IV 39.3% in matched overall cohort | Non-pCR 186/262 (71.0%); the meta-analysis used the non-pCR subgroup |
| Feng et al, 2024 | AIT | 39 | Reported ypT+N+ subgroup | OS 67; DFS/RFS 67 | ESCC 100%; adenocarcinoma 0% | Upper: 12.8% vs 10.9%; Middle: 35.9% vs 50.0%; Lower: 51.3% vs 39.1% | NR | ypT3-4 by definition (ypT+N+) | ypN+ by definition (ypT+N+) | NR | Meta-analysis estimate reconstructed from residual-disease curves |
| Feng et al, 2024 | Control | 46 | Reported ypT+N+ subgroup | OS 83; DFS/RFS 83 | ESCC 100%; adenocarcinoma 0% | Upper: 12.8% vs 10.9%; Middle: 35.9% vs 50.0%; Lower: 51.3% vs 39.1% | NR | ypT3-4 by definition (ypT+N+) | ypN+ by definition (ypT+N+) | NR | Meta-analysis estimate reconstructed from residual-disease curves |
| Geng et al, 2026 | AIT | 126 | non-pCR subgroup (after sIPTW) | OS 126; DFS/RFS 126 | ESCC 100%; adenocarcinoma 0% | Upper: 4.0% vs 3.5%; Middle: 33.9% vs 34.4%; Lower: 62.1% vs 62.1% (after sIPTW) | cStage II: 37.7% vs 36.2%; III: 55.0% vs 57.3%; IV: 7.3% vs 6.4% (after sIPTW) | ypT0: 6.3% vs 7.1%; ypT1: 32.7% vs 34.5%; ypT2: 18.1% vs 17.2%; ypT3: 35.3% vs 35.7%; ypT4: 1.3% vs 0.5% (after sIPTW) | ypN0: 60.3% vs 61.1%; ypN1: 30.3% vs 29.6%; ypN2: 7.4% vs 6.7%; ypN3: 2.0% vs 2.6% (after sIPTW) | pStage I: 42.7% vs 44.4%; II: 16.7% vs 16.7%; III: 37.7% vs 35.8%; IV: 2.9% vs 3.1% (after sIPTW) | The meta-analysis used the non-pCR subgroup |
| Geng et al, 2026 | Control | 98 | non-pCR subgroup (after sIPTW) | OS 98; DFS/RFS 98 | ESCC 100%; adenocarcinoma 0% | Upper: 4.0% vs 3.5%; Middle: 33.9% vs 34.4%; Lower: 62.1% vs 62.1% (after sIPTW) | cStage II: 37.7% vs 36.2%; III: 55.0% vs 57.3%; IV: 7.3% vs 6.4% (after sIPTW) | ypT0: 6.3% vs 7.1%; ypT1: 32.7% vs 34.5%; ypT2: 18.1% vs 17.2%; ypT3: 35.3% vs 35.7%; ypT4: 1.3% vs 0.5% (after sIPTW) | ypN0: 60.3% vs 61.1%; ypN1: 30.3% vs 29.6%; ypN2: 7.4% vs 6.7%; ypN3: 2.0% vs 2.6% (after sIPTW) | pStage I: 42.7% vs 44.4%; II: 16.7% vs 16.7%; III: 37.7% vs 35.8%; IV: 2.9% vs 3.1% (after sIPTW) | The meta-analysis used the non-pCR subgroup |
| Huang et al, 2026 | AIT | 283 | Overall post-NCIT cohort | DFS/RFS 86 | ESCC 100%; adenocarcinoma 0% | Upper 31/283 (11.0%); middle 153/283 (54.1%); lower 99/283 (35.0%) | NR | ypT0 91/283 (32.2%); ypT1 25/283 (8.8%); ypT2 44/283 (15.5%); ypT3 117/283 (41.3%); ypT4a 6/283 (2.1%) | ypN0 133/283 (47.0%); ypN1 96/283 (33.9%); ypN2 40/283 (14.1%); ypN3 14/283 (4.9%) | NR | pCR 64/283 (22.6%); MPR 148/283 (52.3%); the DFS/RFS estimate was derived from residual-risk pathological subgroups |
| Huang et al, 2026 | Control | 283 | Overall post-NCIT cohort | DFS/RFS 43 | ESCC 100%; adenocarcinoma 0% | Upper 31/283 (11.0%); middle 153/283 (54.1%); lower 99/283 (35.0%) | NR | ypT0 91/283 (32.2%); ypT1 25/283 (8.8%); ypT2 44/283 (15.5%); ypT3 117/283 (41.3%); ypT4a 6/283 (2.1%) | ypN0 133/283 (47.0%); ypN1 96/283 (33.9%); ypN2 40/283 (14.1%); ypN3 14/283 (4.9%) | NR | pCR 64/283 (22.6%); MPR 148/283 (52.3%); the DFS/RFS estimate was derived from residual-risk pathological subgroups |

**Supplementary Table S2. Characteristics of the included study populations: Part B (continued)**

| **Study** | **Arm** | **Baseline N** | **Baseline population** | **Meta-analysis population** | **Histology** | **Tumor location** | **Clinical stage** | **ypT stage** | **ypN stage** | **Pathologic stage** | **Residual disease/pCR** |
| --- | --- | --- | --- | --- | --- | --- | --- | --- | --- | --- | --- |
| Kelly et al, 2021/2025 | AIT | 532 | ITT: non-pCR post-NCRT+R0 RCT | OS 532; DFS/RFS 532 | ESCC 29% (nivo) / 29% (placebo); adenocarcinoma 71% (nivo) / 71% (placebo) | Esophagus: 58% vs 58%; GEJ: 42% vs 42% | cStage II: 34% vs 38%; III: 66% vs 62% | ypT0: 6% vs 6%; ypT1-2: 38% vs 40%; ypT3-4: 56% vs 53% | ypN0: 43% vs 42%; ypN+: 57% vs 58% | NR | Residual pathologic disease was required |
| Kelly et al, 2021/2025 | Control | 262 | ITT: non-pCR post-NCRT+R0 RCT | OS 262; DFS/RFS 262 | ESCC 29% (nivo) / 29% (placebo); adenocarcinoma 71% (nivo) / 71% (placebo) | Esophagus: 58% vs 58%; GEJ: 42% vs 42% | cStage II: 34% vs 38%; III: 66% vs 62% | ypT0: 6% vs 6%; ypT1-2: 38% vs 40%; ypT3-4: 56% vs 53% | ypN0: 43% vs 42%; ypN+: 57% vs 58% | NR | Residual pathologic disease was required |
| Koga et al, 2025 | AIT | 15 | Non-pCR population after neoadjuvant chemotherapy and esophagectomy | OS 15; DFS/RFS 15 | ESCC 100%; adenocarcinoma 0% | Ut/Mt/Lt: 0%/40%/60% vs 0%/57.9%/42.1% | cStage II 20%/III 80% vs II 18.4%/III 81.6% | ypT0: 6.7% vs 0%; ypT1: 13.3% vs 13.2%; ypT2: 26.7% vs 21.1%; ypT3: 53.3% vs 65.8% | ypN0: 40.0% vs 44.7%; ypN1: 40.0% vs 39.5%; ypN2: 20.0% vs 13.2%; ypN3: 0% vs 2.6% | NR | Non-pCR population by eligibility; pCR 0% |
| Koga et al, 2025 | Control | 38 | Non-pCR population after neoadjuvant chemotherapy and esophagectomy | OS 38; DFS/RFS 38 | ESCC 100%; adenocarcinoma 0% | Ut/Mt/Lt: 0%/40%/60% vs 0%/57.9%/42.1% | cStage II 20%/III 80% vs II 18.4%/III 81.6% | ypT0: 6.7% vs 0%; ypT1: 13.3% vs 13.2%; ypT2: 26.7% vs 21.1%; ypT3: 53.3% vs 65.8% | ypN0: 40.0% vs 44.7%; ypN1: 40.0% vs 39.5%; ypN2: 20.0% vs 13.2%; ypN3: 0% vs 2.6% | NR | Non-pCR population by eligibility; pCR 0% |
| Liu C et al, 2025 | AIT | 23 | non-pCR cohort (three-group comparison) | OS 23; DFS/RFS 21 | ESCC 78.3% (IO) / 95.6% (no adj) / 75.0% (adj chemo); adenocarcinoma 21.7% (IO) / 4.4% (no adj) / 25.0% (adj chemo) | Upper: 13.0% vs 23.5% (no adj); Middle: 30.4% vs 32.4%; Lower: 56.5% vs 44.1% | cStage II: 8.7% vs 22.1% (no adj); III: 73.9% vs 54.4%; IV: 17.4% vs 20.6% | ypT0: 8.7% vs 16.2% (no adj); ypT1: 17.4% vs 30.9%; ypT2: 4.3% vs 25.0%; ypT3: 65.2% vs 22.1%; ypT4: 4.3% vs 5.9% | ypN0: 39.1% vs 73.5% (no adj); ypN1: 43.5% vs 17.6%; ypN2: 8.7% vs 5.9%; ypN3: 8.7% vs 2.9% | NR | 0% (non-pCR only) |
| Liu C et al, 2025 | Control | 68 | non-pCR cohort (three-group comparison) | OS 68; DFS/RFS 62 | ESCC 78.3% (IO) / 95.6% (no adj) / 75.0% (adj chemo); adenocarcinoma 21.7% (IO) / 4.4% (no adj) / 25.0% (adj chemo) | Upper: 13.0% vs 23.5% (no adj); Middle: 30.4% vs 32.4%; Lower: 56.5% vs 44.1% | cStage II: 8.7% vs 22.1% (no adj); III: 73.9% vs 54.4%; IV: 17.4% vs 20.6% | ypT0: 8.7% vs 16.2% (no adj); ypT1: 17.4% vs 30.9%; ypT2: 4.3% vs 25.0%; ypT3: 65.2% vs 22.1%; ypT4: 4.3% vs 5.9% | ypN0: 39.1% vs 73.5% (no adj); ypN1: 43.5% vs 17.6%; ypN2: 8.7% vs 5.9%; ypN3: 8.7% vs 2.9% | NR | 0% (non-pCR only) |
| Park et al, 2022 | AIT | 45 | ITT: all post-NCRT+surgery (Phase II RCT) | OS 31; DFS/RFS 31 | ESCC 100%; adenocarcinoma 0% | Upper: 6.7% vs 7.3%; Mid-lower: NR | cStage II: 11.1% vs 12.2%; III: 88.9% vs 87.8% | ypT0: 31.1% vs 34.1%; ypT1: 17.8% vs 12.2%; ypT2: 22.2% vs 29.3%; ypT3: 28.9% vs 24.4% | ypN0: 53.3% vs 56.1%; ypN1: 40.0% vs 34.1%; ypN2: 6.7% vs 7.3%; ypN3: 0% vs 2.4% | NR | The meta-analysis used the non-ypCR subgroup |
| Park et al, 2022 | Control | 41 | ITT: all post-NCRT+surgery (Phase II RCT) | OS 27; DFS/RFS 27 | ESCC 100%; adenocarcinoma 0% | Upper: 6.7% vs 7.3%; Mid-lower: NR | cStage II: 11.1% vs 12.2%; III: 88.9% vs 87.8% | ypT0: 31.1% vs 34.1%; ypT1: 17.8% vs 12.2%; ypT2: 22.2% vs 29.3%; ypT3: 28.9% vs 24.4% | ypN0: 53.3% vs 56.1%; ypN1: 40.0% vs 34.1%; ypN2: 6.7% vs 7.3%; ypN3: 0% vs 2.4% | NR | The meta-analysis used the non-ypCR subgroup |

**Supplementary Table S2. Characteristics of the included study populations: Part B (continued)**

| **Study** | **Arm** | **Baseline N** | **Baseline population** | **Meta-analysis population** | **Histology** | **Tumor location** | **Clinical stage** | **ypT stage** | **ypN stage** | **Pathologic stage** | **Residual disease/pCR** |
| --- | --- | --- | --- | --- | --- | --- | --- | --- | --- | --- | --- |
| Sakowitz et al, 2026 | AIT | 342 | non-pCR (ypT+ or ypN+) NCDB cohort | OS 342 | ESCC 12% (IO) / 15% (no-IO); adenocarcinoma 88% (IO) / 85% (no-IO) | Esophagus: 83% vs 82%; GEJ: 17% vs 18% | cStage II: 17% vs 20%; III: 83% vs 80% | ypT0/Tis: 5% vs 8%; ypT1: 21% vs 27%; ypT2: 23% vs 22%; ypT3: 51% vs 42% | ypN0: 53% vs 58%; ypN1: 28% vs 28%; ypN2: 15% vs 10%; ypN3: 4% vs 3% | pStage I: 24% vs 30%; II: 28% vs 24%; III: 40% vs 36%; IVA: 5% vs 3% | Residual pathologic disease, ypT+ or ypN+, was required |
| Sakowitz et al, 2026 | Control | 1025 | non-pCR (ypT+ or ypN+) NCDB cohort | OS 1025 | ESCC 12% (IO) / 15% (no-IO); adenocarcinoma 88% (IO) / 85% (no-IO) | Esophagus: 83% vs 82%; GEJ: 17% vs 18% | cStage II: 17% vs 20%; III: 83% vs 80% | ypT0/Tis: 5% vs 8%; ypT1: 21% vs 27%; ypT2: 23% vs 22%; ypT3: 51% vs 42% | ypN0: 53% vs 58%; ypN1: 28% vs 28%; ypN2: 15% vs 10%; ypN3: 4% vs 3% | pStage I: 24% vs 30%; II: 28% vs 24%; III: 40% vs 36%; IVA: 5% vs 3% | Residual pathologic disease, ypT+ or ypN+, was required |
| Sugase et al, 2025 | AIT | 60 | ypN-positive residual disease after neoadjuvant chemotherapy and surgery; pCR excluded by definition | OS 60; DFS/RFS 60 | ESCC 100%; adenocarcinoma 0% | Ut/Mt/Lt: NR | NR | ypT3: 62% vs 57%; ypT4: NR | ypN+: 100% in both (inclusion criterion) | NR | ypN-positive population by eligibility; ypT0N0 pCR 0% |
| Sugase et al, 2025 | Control | 60 | ypN-positive residual disease after neoadjuvant chemotherapy and surgery; pCR excluded by definition | OS 60; DFS/RFS 60 | ESCC 100%; adenocarcinoma 0% | Ut/Mt/Lt: NR | NR | ypT3: 62% vs 57%; ypT4: NR | ypN+: 100% in both (inclusion criterion) | NR | ypN-positive population by eligibility; ypT0N0 pCR 0% |
| Verhoeven et al, 2026 | AIT | 308 | Residual disease after NCRT and surgery; complete pathologic response (ypT0N0) excluded | OS 308 | ESCC 10% (both groups); adenocarcinoma 90% (both groups) | Mid/distal: 97.1% vs 96.4%; GEJ: NR | cStage II: 18.2% vs 16.6%; III: 81.8% vs 83.4% | ypT0: 3.9%; ypT1: 22.7%; ypT2: 24.7%; ypT3: 47.7%; ypT4: 1.0% | ypN0: 46.4%; ypN1: 31.8%; ypN2: 15.3%; ypN3: 6.5% | NR | Complete pathologic response (ypT0N0) excluded; pCR 0% |
| Verhoeven et al, 2026 | Control | 308 | Residual disease after NCRT and surgery; complete pathologic response (ypT0N0) excluded | OS 308 | ESCC 10% (both groups); adenocarcinoma 90% (both groups) | Mid/distal: 97.1% vs 96.4%; GEJ: NR | cStage II: 18.2% vs 16.6%; III: 81.8% vs 83.4% | ypT0: 3.9%; ypT1: 22.7%; ypT2: 22.1%; ypT3: 50.6%; ypT4: 0.6% | ypN0: 47.7%; ypN1: 29.5%; ypN2: 15.3%; ypN3: 7.5% | NR | Complete pathologic response (ypT0N0) excluded; pCR 0% |
| Wang et al, 2026 | AIT | NR | NPCR classification cohort | OS 94; DFS/RFS 94 | ESCC 100%; adenocarcinoma 0% | Upper: 11.5%; Middle: 38.3%; Lower: 50.2% (overall) | cStage II: 9.9%; III: 70.8%; IV: 19.3% (overall) | ypT0: 18.8% (NPCR); ypT1: 26.1%; ypT2: 22.2%; ypT3-4: 33.0% (NPCR) | ypN0: 31.8%; ypN1: 36.4%; ypN2: 23.3%; ypN3: 8.5% (NPCR) | pStage I: 17.4%; II: 21.6%; III: 39.5%; IV: 7.8% (NPCR) | NPCR/residual-disease subgroup |
| Wang et al, 2026 | Control | NR | NPCR classification cohort | OS 82; DFS/RFS 82 | ESCC 100%; adenocarcinoma 0% | Upper: 11.5%; Middle: 38.3%; Lower: 50.2% (overall) | cStage II: 9.9%; III: 70.8%; IV: 19.3% (overall) | ypT0: 18.8% (NPCR); ypT1: 26.1%; ypT2: 22.2%; ypT3-4: 33.0% (NPCR) | ypN0: 31.8%; ypN1: 36.4%; ypN2: 23.3%; ypN3: 8.5% (NPCR) | pStage I: 17.4%; II: 21.6%; III: 39.5%; IV: 7.8% (NPCR) | NPCR/residual-disease subgroup |

*Baseline N and characteristics refer to the population identified in the Baseline population column; endpoint-specific meta-analysis sample sizes are shown separately. pCR was defined as ypT0N0 when reported.*

*Abbreviations: AIT, adjuvant immunotherapy; ESCC, esophageal squamous cell carcinoma; NR, not reported; pCR, pathologic complete response.*
