# Supplementary material for: Adjuvant immunotherapy versus observation after neoadjuvant therapy and esophagectomy for esophageal cancer with residual pathologic disease: a systematic review and reconstructed individual participant data meta-analysis
Source: Front Immunol. 2026 Sep 24;17:1944032. doi: 10.3389/fimmu.2026.1944032 (PMC13646074; doi:10.3389/fimmu.2026.1944032)
Supplement: Supplementary file 3 [file Table3.docx]

**Table S3. Hazard-ratio sources and analysis populations**

| **Study** | **Outcome** | **Paradigm** | **Analysis population** | **Analyzed N** | **Comparison** | **HR (95% CI)** | **HR source** | **Model** |
| --- | --- | --- | --- | --- | --- | --- | --- | --- |
| Chen et al, 2025 | DFS/RFS | NCIT | Predominantly residual-disease post-NCIT R0 ESCC cohort; ypT0 occurred in 16/169 patients (9.5%), while strict ypT0N0 pCR was not reported | Control 87; AIT 82 | NCIT+S+AIT vs NCIT+S+Ob | 0.7 (0.41-1.21) | Article-reported | Reported Cox model |
| Chen et al, 2026 | DFS/RFS | NCIT | Non-pCR subgroup after NCIT and R0 resection | Control 186; AIT 221 | NCIT+S+AIT vs NCIT+S+Ob | 0.88 (0.61-1.28) | Article-reported | Adjusted Cox model |
| Feng et al, 2024 | DFS/RFS | NCIT | Residual-disease patients with extractable AIT vs observation survival curves | Control 83; AIT 67 | NCIT+S+AIT vs NCIT+S+Ob | 0.69 (0.428-1.11) | KM-reconstructed | Cox model from reconstructed IPD |
| Geng et al, 2026 | DFS/RFS | NCIT | Non-pCR subgroup after NCIT and surgery | Control 98; AIT 126 | NCIT+S+AIT vs NCIT+S+Ob | 0.827 (0.519-1.32) | KM-reconstructed | Cox model from reconstructed IPD |
| Huang et al, 2026 | DFS/RFS | NCIT | Pathological residual-risk subgroup with extractable RFS curves | Control 43; AIT 86 | NCIT+S+AIT vs NCIT+S+Ob | 0.308 (0.165-0.575) | KM-reconstructed | Cox model from reconstructed IPD |
| Kelly et al, 2021/2025 | DFS/RFS | NCRT | Residual pathologic disease after NCRT and R0 resection | Control 262; AIT 532 | NCRT+S+AIT vs NCRT+S+Pbo | 0.76 (0.63-0.91) | Article-reported | Reported Cox model |
| Koga et al, 2025 | DFS/RFS | NCT | Non-pCR population after neoadjuvant chemotherapy and esophagectomy | Control 38; AIT 15 | NCT+S+AIT vs NCT+S+Ob | 0.23 (0.05-1.01) | Article-reported | Reported Cox model |
| Liu C et al, 2025 | DFS/RFS | Mixed NAT | Non-pCR patients after mixed neoadjuvant therapy and surgery | Control 62; AIT 21 | Mixed NAT+S+AIT vs Mixed NAT+S+Ob | 1.33 (0.67-2.67) | KM-reconstructed | Cox model from reconstructed IPD |
| Park et al, 2022 | DFS/RFS | NCRT | Non-ypCR subgroup after NCRT and surgery | Control 27; AIT 31 | NCRT+S+AIT vs NCRT+S+Pbo | 1.02 (0.49-2.13) | Article-reported | Subgroup Cox model |
| Sugase et al, 2025 | DFS/RFS | NCT | ypN-positive residual disease after neoadjuvant chemotherapy and surgery; pCR excluded by definition | Control 60; AIT 60 | NCT+S+AIT vs NCT+S+Ob | 0.49 (0.28-0.83) | Article-reported | Adjusted Cox model |
| Wang et al, 2026 | DFS/RFS | NCIT | NPCR/residual-disease subgroup after NCIT and surgery | Control 82; AIT 94 | NCIT+S+AIT vs NCIT+S+Ob | 0.82 (0.53-1.28) | Article-reported | Subgroup Cox model |

**Table S3. Hazard-ratio sources and analysis populations (continued)**

| **Study** | **Outcome** | **Paradigm** | **Analysis population** | **Analyzed N** | **Comparison** | **HR (95% CI)** | **HR source** | **Model** |
| --- | --- | --- | --- | --- | --- | --- | --- | --- |
| Chen et al, 2025 | OS | NCIT | Predominantly residual-disease post-NCIT R0 ESCC cohort; ypT0 occurred in 16/169 patients (9.5%), while strict ypT0N0 pCR was not reported | Control 87; AIT 82 | NCIT+S+AIT vs NCIT+S+Ob | 0.53 (0.28-0.99) | Article-reported | Reported Cox model |
| Chen et al, 2026 | OS | NCIT | Non-pCR subgroup after NCIT and R0 resection | Control 186; AIT 221 | NCIT+S+AIT vs NCIT+S+Ob | 0.72 (0.48-0.84) | Article-reported | Adjusted Cox model |
| Feng et al, 2024 | OS | NCIT | Residual-disease patients with extractable AIT vs observation survival curves | Control 83; AIT 67 | NCIT+S+AIT vs NCIT+S+Ob | 0.562 (0.316-0.999) | KM-reconstructed | Cox model from reconstructed IPD |
| Geng et al, 2026 | OS | NCIT | Non-pCR subgroup after NCIT and surgery | Control 98; AIT 126 | NCIT+S+AIT vs NCIT+S+Ob | 0.369 (0.175-0.777) | KM-reconstructed | Cox model from reconstructed IPD |
| Kelly et al, 2021/2025 | OS | NCRT | Residual pathologic disease after NCRT and R0 resection | Control 262; AIT 532 | NCRT+S+AIT vs NCRT+S+Pbo | 0.85 (0.7-1.04) | Article-reported | Reported Cox model |
| Koga et al, 2025 | OS | NCT | Non-pCR population after neoadjuvant chemotherapy and esophagectomy | Control 38; AIT 15 | NCT+S+AIT vs NCT+S+Ob | 0.47 (0.09-2.33) | Article-reported | Reported Cox model |
| Liu C et al, 2025 | OS | Mixed NAT | Non-pCR patients after mixed neoadjuvant therapy and surgery | Control 68; AIT 23 | Mixed NAT+S+AIT vs Mixed NAT+S+Ob | 1.16 (0.561-2.38) | KM-reconstructed | Cox model from reconstructed IPD |
| Park et al, 2022 | OS | NCRT | Non-ypCR subgroup after NCRT and surgery | Control 27; AIT 31 | NCRT+S+AIT vs NCRT+S+Pbo | 0.79 (0.34-1.81) | Article-reported | Subgroup Cox model |
| Sakowitz et al, 2026 | OS | NCRT | Residual pathologic disease after NCRT and esophagectomy | Control 1025; AIT 342 | NCRT+S+AIT vs NCRT+S+Ob | 0.6 (0.44-0.81) | Article-reported | Adjusted Cox model |
| Sugase et al, 2025 | OS | NCT | ypN-positive residual disease after neoadjuvant chemotherapy and surgery; pCR excluded by definition | Control 60; AIT 60 | NCT+S+AIT vs NCT+S+Ob | 0.17 (0.06-0.5) | Article-reported | Adjusted Cox model |
| Verhoeven et al, 2026 | OS | NCRT | Residual disease after NCRT and surgery; complete pathologic response (ypT0N0) excluded | Control 308; AIT 308 | NCRT+S+AIT vs NCRT+S+Ob | 0.75 (0.6-0.97) | Article-reported | Adjusted Cox model |
| Wang et al, 2026 | OS | NCIT | NPCR/residual-disease subgroup after NCIT and surgery | Control 82; AIT 94 | NCIT+S+AIT vs NCIT+S+Ob | 0.34 (0.17-0.7) | Article-reported | Subgroup Cox model |

*Article-reported HRs were used when they matched the eligible comparison and analysis population. When no suitable reported HR was available, a Cox HR was estimated from reconstructed Kaplan-Meier individual participant data.*

*Abbreviations: AIT, adjuvant immunotherapy; CI, confidence interval; DFS, disease-free survival; HR, hazard ratio; IPD, individual participant data; NCIT, neoadjuvant chemoimmunotherapy; NCRT, neoadjuvant chemoradiotherapy; NCT, neoadjuvant chemotherapy; OS, overall survival; RFS, recurrence-free survival.*
