# Supplementary material for: Adjuvant immunotherapy versus observation after neoadjuvant therapy and esophagectomy for esophageal cancer with residual pathologic disease: a systematic review and reconstructed individual participant data meta-analysis
Source: Front Immunol. 2026 Sep 24;17:1944032. doi: 10.3389/fimmu.2026.1944032 (PMC13646074; doi:10.3389/fimmu.2026.1944032)
Supplement: Supplementary file 4 [file Table4.docx]

**Table S4. Risk-of-bias assessment of included studies**

**A. Randomized trials: Cochrane Risk of Bias 2**

| **Study** | **Design** | **D1** | **D2** | **D3** | **D4** | **D5** | **Overall judgment** |
| --- | --- | --- | --- | --- | --- | --- | --- |
| Kelly et al, 2021/2025 | Phase 3 randomized trial | Low | Low | Low | Low | Low | Low |
| Park et al, 2022 | Phase 2 randomized trial | Low | Low | Low | Low | Some concerns | Some concerns |

**B. Observational studies: Newcastle-Ottawa Scale**

| **Study** | **Design** | **Selection (0-4)** | **Comparability (0-2)** | **Outcome (0-3)** | **Total (0-9)** | **Overall judgment** |  |
| --- | --- | --- | --- | --- | --- | --- | --- |
| Chen et al, 2025 | Retrospective cohort | 3 | 1 | 2 | 6 | Moderate |  |
| Chen et al, 2026 | Retrospective cohort | 3 | 2 | 3 | 8 | Low |  |
| Feng et al, 2024 | Retrospective cohort | 3 | 1 | 2 | 6 | Moderate |  |
| Geng et al, 2026 | Retrospective cohort | 3 | 2 | 2 | 7 | Moderate |  |
| Huang et al, 2026 | Retrospective cohort | 3 | 2 | 2 | 7 | Moderate |  |
| Koga et al, 2025 | Retrospective cohort | 2 | 1 | 2 | 5 | High |  |
| Liu C et al, 2025 | Retrospective cohort | 3 | 1 | 2 | 6 | Moderate |  |
| Sakowitz et al, 2026 | Retrospective registry cohort | 3 | 2 | 2 | 7 | Moderate |  |
| Sugase et al, 2025 | Retrospective cohort | 2 | 2 | 2 | 6 | Moderate |  |
| Verhoeven et al, 2026 | Retrospective registry cohort | 4 | 2 | 2 | 8 | Low |  |
| Wang et al, 2026 | Retrospective cohort | 3 | 1 | 2 | 6 | Moderate |  |

*RoB 2 domains: D1, bias arising from the randomization process; D2, bias due to deviations from intended interventions; D3, bias due to missing outcome data; D4, bias in measurement of the outcome; D5, bias in selection of the reported result.*

*The Park et al, 2022 judgment pertains to the article-reported non-ypCR subgroup HR included in this meta-analysis. Randomization, masking, outcome completeness, and outcome measurement were judged low risk; D5 was rated as some concerns because the subgroup result was not the primary intention-to-treat estimate and prespecification was not clearly documented.*

*For the Newcastle-Ottawa Scale, higher scores indicate lower risk of bias. Overall judgments used prespecified thresholds: 8-9, low risk; 6-7, moderate risk; 0-5, high risk.*
