# Supplementary material for: Adjuvant immunotherapy versus observation after neoadjuvant therapy and esophagectomy for esophageal cancer with residual pathologic disease: a systematic review and reconstructed individual participant data meta-analysis
Source: Front Immunol. 2026 Sep 24;17:1944032. doi: 10.3389/fimmu.2026.1944032 (PMC13646074; doi:10.3389/fimmu.2026.1944032)
Supplement: Supplementary file 5 [file Table5.docx]

**Table S5. Sensitivity analyses, alternative estimators, and small-study effects**

**A. Primary sensitivity analyses**

| **Outcome** | **Analysis** | **Studies, No.** | **Participants, No.** | **Studies excluded** | **REML HR (95% CI)** | **Knapp-Hartung HR (95% CI)** | **95% prediction interval** | **I², %** | **τ²** | **P for heterogeneity** |
| --- | --- | --- | --- | --- | --- | --- | --- | --- | --- | --- |
| DFS/RFS | Primary analysis | 11 | 2363 | None | 0.74 (0.66-0.84) | 0.74 (0.62-0.89) | 0.66-0.84 | 41.4 | 0.000 | .073 |
| DFS/RFS | Strict residual-disease cohorts | 10 | 2194 | Chen et al, 2025 | 0.73 (0.60-0.88) | 0.73 (0.56-0.95) | 0.49-1.09 | 47.1 | 0.032 | .048 |
| DFS/RFS | Excluding KM-reconstructed HRs | 7 | 1777 | Feng et al, 2024; Geng et al, 2026; Huang et al, 2026; Liu C et al, 2025 | 0.76 (0.66-0.87) | 0.76 (0.63-0.90) | 0.66-0.87 | 5.5 | 0.000 | .385 |
| DFS/RFS | Reported adjusted, weighted, or randomized HRs only | 4 | 1379 | Chen et al, 2025; Feng et al, 2024; Geng et al, 2026; Huang et al, 2026; Koga et al, 2025; Liu C et al, 2025; Wang et al, 2026 | 0.76 (0.65-0.89) | 0.76 (0.58-1.01) | 0.65-0.89 | 19.4 | 0.000 | .293 |
| DFS/RFS | Excluding national database cohorts | 11 | 2363 | None | 0.74 (0.66-0.84) | 0.74 (0.62-0.89) | 0.66-0.84 | 41.4 | 0.000 | .073 |
| DFS/RFS | Excluding influential or outlying studies | 7 | 913 | Chen et al, 2026; Huang et al, 2026; Kelly et al, 2021/2025; Sugase et al, 2025 | 0.80 (0.65-1.00) | 0.80 (0.62-1.05) | 0.65-1.00 | 0.0 | 0.000 | .449 |
| OS | Primary analysis | 12 | 4225 | None | 0.64 (0.53-0.77) | 0.64 (0.51-0.81) | 0.41-0.99 | 50.4 | 0.041 | .023 |
| OS | Strict residual-disease cohorts | 11 | 4056 | Chen et al, 2025 | 0.65 (0.53-0.79) | 0.65 (0.50-0.84) | 0.41-1.03 | 53.2 | 0.045 | .019 |
| OS | Excluding KM-reconstructed HRs | 9 | 3760 | Feng et al, 2024; Geng et al, 2026; Liu C et al, 2025 | 0.65 (0.54-0.79) | 0.65 (0.50-0.85) | 0.43-0.99 | 52.4 | 0.035 | .032 |
| OS | Reported adjusted, weighted, or randomized HRs only | 6 | 3362 | Chen et al, 2025; Feng et al, 2024; Geng et al, 2026; Koga et al, 2025; Liu C et al, 2025; Wang et al, 2026 | 0.72 (0.62-0.84) | 0.72 (0.55-0.95) | 0.57-0.92 | 55.2 | 0.009 | .048 |
| OS | Excluding national database cohorts | 10 | 2242 | Sakowitz et al, 2026; Verhoeven et al, 2026 | 0.59 (0.44-0.79) | 0.59 (0.42-0.84) | 0.30-1.18 | 56.7 | 0.103 | .014 |
| OS | Excluding influential or outlying studies | 9 | 2904 | Chen et al, 2026; Kelly et al, 2021/2025; Sugase et al, 2025 | 0.62 (0.51-0.76) | 0.62 (0.49-0.79) | 0.45-0.87 | 23.8 | 0.019 | .232 |

HRs below 1 favor adjuvant immunotherapy.

**B. Alternative meta-analytic estimators**

| **Outcome** | **Estimator** | **Studies, No.** | **Model** | **HR (95% CI)** | **95% prediction interval** | **Prediction interval includes no effect** | **I², %** | **τ²** | **P for heterogeneity** |
| --- | --- | --- | --- | --- | --- | --- | --- | --- | --- |
| DFS/RFS | Fixed-effect | 11 | FE/z | 0.74 (0.66-0.84) | Not applicable | Not applicable | 41.4 | 0.000 | .073 |
| DFS/RFS | DerSimonian-Laird random-effects | 11 | DL/z | 0.72 (0.60-0.88) | 0.47-1.11 | Yes | 41.4 | 0.038 | .073 |
| DFS/RFS | REML random-effects | 11 | REML/z | 0.74 (0.66-0.84) | 0.66-0.84 | No | 41.4 | 0.000 | .073 |
| DFS/RFS | REML with Knapp-Hartung | 11 | REML/knha | 0.74 (0.62-0.89) | 0.62-0.89 | No | 41.4 | 0.000 | .073 |
| DFS/RFS | Paule-Mandel random-effects | 11 | PM/z | 0.72 (0.57-0.90) | 0.39-1.30 | Yes | 41.4 | 0.079 | .073 |
| DFS/RFS | Sidik-Jonkman random-effects | 11 | SJ/z | 0.71 (0.54-0.94) | 0.32-1.55 | Yes | 41.4 | 0.139 | .073 |
| OS | Fixed-effect | 12 | FE/z | 0.71 (0.63-0.79) | Not applicable | Not applicable | 50.4 | 0.000 | .023 |
| OS | DerSimonian-Laird random-effects | 12 | DL/z | 0.64 (0.53-0.77) | 0.40-1.01 | Yes | 50.4 | 0.045 | .023 |
| OS | REML random-effects | 12 | REML/z | 0.64 (0.53-0.77) | 0.41-0.99 | No | 50.4 | 0.041 | .023 |
| OS | REML with Knapp-Hartung | 12 | REML/knha | 0.64 (0.51-0.81) | 0.39-1.06 | Yes | 50.4 | 0.041 | .023 |
| OS | Paule-Mandel random-effects | 12 | PM/z | 0.62 (0.49-0.78) | 0.33-1.16 | Yes | 50.4 | 0.088 | .023 |
| OS | Sidik-Jonkman random-effects | 12 | SJ/z | 0.60 (0.46-0.79) | 0.27-1.34 | Yes | 50.4 | 0.146 | .023 |

**C. Leave-one-out analyses**

| **Outcome** | **Omitted study** | **HR source** | **Pooled HR (95% CI)** | **I², %** | **τ²** | **P for heterogeneity** | **Relative HR change, %** |
| --- | --- | --- | --- | --- | --- | --- | --- |
| DFS/RFS | Chen et al, 2025 | Reported HR | 0.73 (0.60-0.88) | 47.1 | 0.032 | .048 | -2.0 |
| DFS/RFS | Chen et al, 2026 | Reported HR | 0.71 (0.58-0.86) | 44.3 | 0.028 | .063 | -4.9 |
| DFS/RFS | Feng et al, 2024 | KM reconstructed | 0.73 (0.60-0.89) | 47.0 | 0.036 | .049 | -1.9 |
| DFS/RFS | Geng et al, 2026 | KM reconstructed | 0.71 (0.59-0.87) | 46.6 | 0.036 | .051 | -3.9 |
| DFS/RFS | Huang et al, 2026 | KM reconstructed | 0.77 (0.68-0.87) | 1.3 | 0.000 | .427 | 3.7 |
| DFS/RFS | Kelly et al, 2021/2025 | Reported HR | 0.71 (0.56-0.90) | 46.9 | 0.057 | .049 | -4.2 |
| DFS/RFS | Koga et al, 2025 | Reported HR | 0.75 (0.66-0.85) | 38.9 | 0.000 | .099 | 0.8 |
| DFS/RFS | Liu C et al, 2025 | KM reconstructed | 0.73 (0.64-0.83) | 36.9 | 0.000 | .114 | -1.9 |
| DFS/RFS | Park et al, 2022 | Reported HR | 0.72 (0.61-0.85) | 44.9 | 0.016 | .060 | -2.9 |
| DFS/RFS | Sugase et al, 2025 | Reported HR | 0.76 (0.67-0.86) | 38.8 | 0.000 | .100 | 2.3 |
| DFS/RFS | Wang et al, 2026 | Reported HR | 0.71 (0.58-0.87) | 46.6 | 0.037 | .051 | -4.0 |
| OS | Chen et al, 2025 | Reported HR | 0.65 (0.53-0.79) | 53.2 | 0.045 | .019 | 0.8 |
| OS | Chen et al, 2026 | Reported HR | 0.61 (0.48-0.77) | 54.8 | 0.069 | .014 | -4.8 |
| OS | Feng et al, 2024 | KM reconstructed | 0.64 (0.52-0.79) | 53.6 | 0.050 | .018 | -0.0 |
| OS | Geng et al, 2026 | KM reconstructed | 0.67 (0.57-0.80) | 47.9 | 0.025 | .038 | 4.8 |
| OS | Kelly et al, 2021/2025 | Reported HR | 0.61 (0.51-0.74) | 41.8 | 0.026 | .071 | -4.4 |
| OS | Koga et al, 2025 | Reported HR | 0.64 (0.53-0.78) | 54.4 | 0.041 | .016 | 0.3 |
| OS | Liu C et al, 2025 | KM reconstructed | 0.62 (0.51-0.75) | 50.8 | 0.045 | .026 | -3.8 |
| OS | Park et al, 2022 | Reported HR | 0.63 (0.51-0.77) | 54.7 | 0.052 | .015 | -2.2 |
| OS | Sakowitz et al, 2026 | Reported HR | 0.63 (0.50-0.79) | 52.2 | 0.066 | .022 | -1.7 |
| OS | Sugase et al, 2025 | Reported HR | 0.68 (0.58-0.80) | 34.0 | 0.018 | .126 | 6.5 |
| OS | Verhoeven et al, 2026 | Reported HR | 0.61 (0.48-0.77) | 54.2 | 0.066 | .016 | -5.4 |
| OS | Wang et al, 2026 | Reported HR | 0.68 (0.58-0.80) | 44.4 | 0.019 | .055 | 6.5 |

Each study was omitted in turn and the primary REML model was refitted.

**D. Small-study effects**

| **Outcome** | **Studies, No.** | **Egger intercept** | **Egger P** | **Begg τ** | **Begg P** | **Trim-and-fill studies, No.** | **Trim-and-fill HR (95% CI)** |
| --- | --- | --- | --- | --- | --- | --- | --- |
| DFS/RFS | 11 | -0.088 | .262 | -0.200 | .445 | 0 | 0.74 (0.66-0.84) |
| OS | 12 | -0.081 | .003 | -0.364 | .116 | 4 | 0.73 (0.56-0.94) |

Funnel asymmetry and regression tests were interpreted as possible small-study effects. Trim-and-fill results are exploratory.

**E. Guarantee-time bias sensitivity analyses**

| **Outcome** | **Analysis** | **Studies included** | **Studies, No.** | **Participants, No.** | **REML HR (95% CI)** | **Knapp-Hartung HR (95% CI)** | **95% prediction interval** | **I², %** | **τ²** | **P for heterogeneity** |
| --- | --- | --- | --- | --- | --- | --- | --- | --- | --- | --- |
| OS | Strictly aligned time zero (descriptive) | Kelly et al, 2021/2025; Park et al, 2022; Verhoeven et al, 2026 | 3 | 1468 | 0.81 (0.69-0.94) | 0.81 (0.67-0.97) | Not estimated | 0.0 | 0.000 | .732 |
| OS | Explicit guarantee-time mitigation | Chen et al, 2025; Geng et al, 2026; Kelly et al, 2021/2025; Park et al, 2022; Sugase et al, 2025; Verhoeven et al, 2026 | 6 | 1981 | 0.59 (0.41-0.86) | 0.59 (0.34-1.02) | 0.27-1.31 | 63.6 | 0.128 | .018 |
| DFS/RFS | Strictly aligned time zero (descriptive) | Kelly et al, 2021/2025; Park et al, 2022 | 2 | 852 | 0.77 (0.65-0.92) | 0.77 (0.32-1.87) | Not estimated | 0.0 | 0.000 | .446 |
| DFS/RFS | Explicit guarantee-time mitigation | Chen et al, 2025; Kelly et al, 2021/2025; Koga et al, 2025; Park et al, 2022; Sugase et al, 2025 | 5 | 1194 | 0.73 (0.62-0.86) | 0.73 (0.59-0.91) | 0.62-0.86 | 0.0 | 0.000 | .461 |
| DFS/RFS | Koga 6-month landmark HR substituted | All primary-analysis studies | 11 | 2363 | 0.75 (0.66-0.84) | 0.75 (0.63-0.89) | 0.66-0.84 | 34.7 | 0.000 | .121 |

Strictly aligned time-zero analyses were descriptive because only 3 OS studies and 2 DFS/RFS studies were eligible; prediction intervals were therefore not reported. Expanded analyses also allowed an endpoint-relevant early-event restriction or landmark analysis.

**F. Sensitivity analyses by postoperative treatment composition**

| **Outcome** | **Analysis** | **Studies, No.** | **Participants, No.** | **REML HR (95% CI)** | **Knapp-Hartung HR (95% CI)** | **95% prediction interval** | **I², %** | **τ²** | **P for heterogeneity** |
| --- | --- | --- | --- | --- | --- | --- | --- | --- | --- |
| OS | Primary analysis | 12 | 4225 | 0.64 (0.53-0.77) | 0.64 (0.51-0.81) | 0.41-0.99 | 50.4 | 0.041 | .023 |
| OS | No substantial or inseparable postoperative chemotherapy | 10 | 2634 | 0.67 (0.55-0.83) | 0.67 (0.51-0.89) | 0.44-1.03 | 49.1 | 0.037 | .039 |
| DFS/RFS | Primary analysis | 11 | 2363 | 0.74 (0.66-0.84) | 0.74 (0.62-0.89) | 0.66-0.84 | 41.4 | 0.000 | .073 |
| DFS/RFS | No substantial or inseparable postoperative chemotherapy | 9 | 2010 | 0.77 (0.67-0.87) | 0.77 (0.65-0.90) | 0.67-0.87 | 11.3 | 0.000 | .341 |

The stricter analysis excluded Geng et al, Huang et al, and Sakowitz et al because postoperative chemotherapy was frequent or could not be separated from the ICI-containing regimen.
