# Supplementary material for: Adjuvant immunotherapy versus observation after neoadjuvant therapy and esophagectomy for esophageal cancer with residual pathologic disease: a systematic review and reconstructed individual participant data meta-analysis
Source: Front Immunol. 2026 Sep 24;17:1944032. doi: 10.3389/fimmu.2026.1944032 (PMC13646074; doi:10.3389/fimmu.2026.1944032)
Supplement: Supplementary file 6 [file Table6.docx]

**Table S6. Bayesian hierarchical meta-analysis and prior-sensitivity analyses**

**A. Primary Bayesian hierarchical models**

| **Outcome** | **Analysis** | **Studies, No.** | **Participants, No.** | **Prior for pooled log(HR)** | **Prior for heterogeneity** | **Bayesian HR (95% CrI)** | **Posterior τ (95% CrI)** | **Pr(HR<1)** | **95% predictive interval** |
| --- | --- | --- | --- | --- | --- | --- | --- | --- | --- |
| OS | Overall | 12 | 4225 | Normal(0, 1) | Half-Normal(0, 0.5) | 0.65 (0.49-0.79) | 0.22 (0.02-0.55) | >99.9% | 0.33-1.14 |
| OS | NCIT | 5 | 1126 | Normal(0, 1) | Half-Normal(0, 0.5) | 0.56 (0.37-0.78) | 0.23 (0.01-0.70) | 99.7% | 0.25-1.15 |
| OS | NCRT | 4 | 2835 | Normal(0, 1) | Half-Normal(0, 0.5) | 0.75 (0.56-1.01) | 0.15 (0.01-0.60) | 97.2% | 0.41-1.38 |
| OS | NCT | 2 | 173 | Normal(0, 1) | Half-Normal(0, 0.5) | 0.33 (0.13-1.00) | 0.34 (0.02-1.09) | 97.5% | 0.09-1.66 |
| OS | Mixed NAT (single study) | 1 | 91 | Normal(0, 1) | Half-Normal(0, 0.5) | 1.12 (0.42-2.91) | 0.31 (0.01-1.05) | 39.9% | 0.27-4.41 |
| DFS/RFS | Overall | 11 | 2363 | Normal(0, 1) | Half-Normal(0, 0.5) | 0.73 (0.59-0.89) | 0.16 (0.01-0.51) | 99.7% | 0.42-1.23 |
| DFS/RFS | NCIT | 6 | 1255 | Normal(0, 1) | Half-Normal(0, 0.5) | 0.71 (0.51-0.96) | 0.21 (0.01-0.66) | 98.5% | 0.34-1.42 |
| DFS/RFS | NCRT | 2 | 852 | Normal(0, 1) | Half-Normal(0, 0.5) | 0.81 (0.48-1.59) | 0.24 (0.01-0.91) | 80.7% | 0.31-2.47 |
| DFS/RFS | NCT | 2 | 173 | Normal(0, 1) | Half-Normal(0, 0.5) | 0.47 (0.22-1.06) | 0.29 (0.01-1.00) | 96.8% | 0.14-1.68 |
| DFS/RFS | Mixed NAT (single study) | 1 | 83 | Normal(0, 1) | Half-Normal(0, 0.5) | 1.26 (0.47-3.17) | 0.31 (0.01-1.06) | 30.3% | 0.30-4.81 |

Rows with one study show a hierarchical-model posterior but do not constitute a pooled subgroup estimate.

**B. Prior-sensitivity analyses**

| **Outcome** | **Prior scenario** | **Studies, No.** | **Prior for pooled log(HR)** | **Prior for heterogeneity** | **Bayesian HR (95% CrI)** | **Posterior τ (95% CrI)** | **Pr(HR<1)** | **95% predictive interval** |
| --- | --- | --- | --- | --- | --- | --- | --- | --- |
| OS | Primary | 12 | Normal(0, 1.00^2) | Half-Normal(0, 0.50^2) | 0.65 (0.49-0.79) | 0.22 (0.02-0.55) | >99.9% | 0.33-1.14 |
| OS | Wider treatment-effect prior | 12 | Normal(0, 2.00^2) | Half-Normal(0, 0.50^2) | 0.65 (0.49-0.78) | 0.22 (0.02-0.56) | >99.9% | 0.32-1.14 |
| OS | Narrower heterogeneity prior | 12 | Normal(0, 1.00^2) | Half-Normal(0, 0.25^2) | 0.66 (0.51-0.78) | 0.18 (0.01-0.43) | >99.9% | 0.38-1.03 |
| OS | Wider heterogeneity prior | 12 | Normal(0, 1.00^2) | Half-Normal(0, 1.00^2) | 0.65 (0.48-0.79) | 0.23 (0.02-0.61) | >99.9% | 0.31-1.19 |
| DFS/RFS | Primary | 11 | Normal(0, 1.00^2) | Half-Normal(0, 0.50^2) | 0.73 (0.59-0.89) | 0.16 (0.01-0.51) | 99.7% | 0.42-1.23 |
| DFS/RFS | Wider treatment-effect prior | 11 | Normal(0, 2.00^2) | Half-Normal(0, 0.50^2) | 0.73 (0.58-0.88) | 0.16 (0.01-0.51) | 99.7% | 0.41-1.23 |
| DFS/RFS | Narrower heterogeneity prior | 11 | Normal(0, 1.00^2) | Half-Normal(0, 0.25^2) | 0.73 (0.60-0.87) | 0.13 (0.01-0.40) | 99.9% | 0.47-1.12 |
| DFS/RFS | Wider heterogeneity prior | 11 | Normal(0, 1.00^2) | Half-Normal(0, 1.00^2) | 0.73 (0.58-0.89) | 0.18 (0.01-0.57) | 99.6% | 0.40-1.29 |

Abbreviations: CrI, credible interval; HR, hazard ratio. τ denotes the between-study standard deviation on the log(HR) scale.
