# Supplementary material for: Adjuvant immunotherapy versus observation after neoadjuvant therapy and esophagectomy for esophageal cancer with residual pathologic disease: a systematic review and reconstructed individual participant data meta-analysis
Source: Front Immunol. 2026 Sep 24;17:1944032. doi: 10.3389/fimmu.2026.1944032 (PMC13646074; doi:10.3389/fimmu.2026.1944032)
Supplement: Supplementary file 7 [file Table7.docx]

**Table S7. GRADE evidence profile for the main and paradigm-stratified analyses**

**A. OS**

| **Outcome** | **Analysis** | **Studies, No.** | **Participants, No.** | **HR (95% CI)** | **Risk of bias** | **Inconsistency** | **Indirectness** | **Imprecision** | **Publication bias** | **Certainty** | **Rationale** |
| --- | --- | --- | --- | --- | --- | --- | --- | --- | --- | --- | --- |
| OS | Main analysis | 12 | 4225 | HR 0.64 (0.53-0.77) | Some concern | Some concern | Some concern | No serious concern | Some concern | Low | Low certainty because observational evidence predominated and heterogeneity was moderate; funnel asymmetry was treated as possible small-study effects, not as definitive publication bias. |
| OS | NCIT | 5 | 1126 | HR 0.54 (0.40-0.73) | Some concern | No serious concern | No serious concern | No serious concern | Not assessable | Low | Low certainty because all NCIT evidence was observational and susceptible to residual confounding despite generally favorable and consistent estimates. |
| OS | NCRT | 4 | 2835 | HR 0.75 (0.63-0.89) | Some concern | No serious concern | No serious concern | No serious concern | Not assessable | Low | Low certainty because randomized evidence has not established an overall-survival benefit and the pooled association depends substantially on observational cohorts. |
| OS | NCT | 2 | 173 | HR 0.23 (0.09-0.58) | Serious concern | No serious concern | No serious concern | Some concern | Not assessable | Very low | Very low certainty because only two small observational studies were available and the estimate was sensitive to sparse data. |
| OS | Mixed NAT | 1 | 91 | HR 1.16 (0.56-2.38) | Serious concern | No serious concern | Serious concern | Serious concern | Not assessable | Very low | Very low certainty because only one clinically heterogeneous cohort remained after excluding a comparison that did not isolate immunotherapy. |

**B. DFS/RFS**

| **Outcome** | **Analysis** | **Studies, No.** | **Participants, No.** | **HR (95% CI)** | **Risk of bias** | **Inconsistency** | **Indirectness** | **Imprecision** | **Publication bias** | **Certainty** | **Rationale** |
| --- | --- | --- | --- | --- | --- | --- | --- | --- | --- | --- | --- |
| DFS/RFS | Main analysis | 11 | 2363 | HR 0.74 (0.66-0.84) | Some concern | Some concern | Some concern | No serious concern | No serious concern | Low | Low certainty because observational evidence predominated, although the pooled estimate was precise and heterogeneity was limited. |
| DFS/RFS | NCIT | 6 | 1255 | HR 0.71 (0.55-0.91) | Some concern | Some concern | No serious concern | No serious concern | Not assessable | Low | Low certainty because all NCIT evidence was observational and susceptible to residual confounding despite generally favorable and consistent estimates. |
| DFS/RFS | NCRT | 2 | 852 | HR 0.77 (0.65-0.92) | Some concern | No serious concern | No serious concern | Some concern | Not assessable | Moderate | Moderate certainty, anchored by randomized placebo-controlled evidence with a precise estimate; downgraded for remaining concerns from the mixed evidence base. |
| DFS/RFS | NCT | 2 | 173 | HR 0.45 (0.27-0.75) | Serious concern | No serious concern | No serious concern | Some concern | Not assessable | Very low | Very low certainty because only two small observational studies were available and the estimate was sensitive to sparse data. |
| DFS/RFS | Mixed NAT | 1 | 83 | HR 1.33 (0.67-2.66) | Serious concern | No serious concern | Serious concern | Serious concern | Not assessable | Very low | Very low certainty because only one clinically heterogeneous cohort remained after excluding a comparison that did not isolate immunotherapy. |

GRADE judgments considered study design, risk of bias, inconsistency, indirectness, imprecision, and possible publication bias. Funnel-plot findings and Egger regression were not used as stand-alone criteria for downgrading certainty.
