# Supplementary material for: Adjuvant immunotherapy versus observation after neoadjuvant therapy and esophagectomy for esophageal cancer with residual pathologic disease: a systematic review and reconstructed individual participant data meta-analysis
Source: Front Immunol. 2026 Sep 24;17:1944032. doi: 10.3389/fimmu.2026.1944032 (PMC13646074; doi:10.3389/fimmu.2026.1944032)
Supplement: Supplementary file 8 [file Table8.docx]

**Table S8. Study-level assessment of potential immortal-time bias**

**A. Survival time origin and timing-specific methods**

| **Study** | **Design** | **OS time origin** | **DFS/RFS time origin** | **Surgery-to-AIT interval** | **Early postoperative events** | **Landmark or aligned index date** | **Time-dependent exposure** |
| --- | --- | --- | --- | --- | --- | --- | --- |
| Chen et al, 2025 | Multicenter retrospective cohort | Diagnosis | Diagnosis | NR | Recurrence or death within 3 months excluded | No formal landmark; 3-month event-free eligibility restriction | No |
| Chen et al, 2026 | Multicenter retrospective cohort | Start of treatment | Surgery | Initial dose typically 4-8 weeks after surgery | None reported | No | No |
| Feng et al, 2024 | Single-center retrospective cohort | NR | NR | NR | Surgery-related mortality excluded; no defined window | No | No |
| Geng et al, 2026 | Multicenter retrospective cohort | NR | NR | Within 3 months after surgery | Required survival beyond 90 days; early recurrence not addressed | No formal landmark; 90-day survivor restriction | No |
| Huang et al, 2026 | Dual-center retrospective cohort | Surgery | Surgery | Within 1-2 months after surgery | None reported | No | No |
| Kelly et al, 2021/2025 | Randomized phase III trial | Randomization | Randomization | Randomization 4-16 weeks after surgery | Eligibility assessed before randomization | Common randomized time zero | Not applicable |
| Koga et al, 2025 | Single-center retrospective cohort | Surgery | Surgery | Median 188 days (IQR, 108-223) | Seven early recurrences in the control group and none in the AIT group | Six-month landmark for DFS only | No |
| Liu C et al, 2025 | Single-center retrospective cohort | Diagnosis | Surgery | NR | None reported | No | No |
| Park et al, 2022 | Randomized phase II trial | Randomization | Randomization | Trial entry 21-56 days after surgery | Eligibility assessed before randomization | Common randomized time zero | Not applicable |
| Sakowitz et al, 2026 | National database cohort | Esophagectomy | Not reported | NR in the NCDB | Ninety-day morbidity, including mortality, used as a covariate | No | No |
| Sugase et al, 2025 | Single-center retrospective cohort | Surgery | Surgery | 45% within 10 weeks; 55% at 10 weeks or later | Recurrence or death within 3 months excluded | No formal landmark; 3-month event-free eligibility restriction | No |
| Verhoeven et al, 2026 | Nationwide registry matched cohort | Actual or matched pseudo-index date | Not reported | Typically more than 2 months after surgery | Death or distant metastasis within 14 weeks excluded | Control index date matched to the treated patient's AIT start interval | No |
| Wang et al, 2026 | Single-center retrospective cohort | Surgery | Surgery | NR | None reported | No | No |

**B. Confounding control and endpoint-specific assessment**

| **Study** | **Conventional confounding control** | **Relevance to guarantee-time bias** | **OS assessment** | **DFS/RFS assessment** |
| --- | --- | --- | --- | --- |
| Chen et al, 2025 | Multivariable Cox regression | Early-event restriction partially addresses timing; Cox adjustment alone does not | Partially addressed | Partially addressed |
| Chen et al, 2026 | Propensity-score matching and Cox regression | Treatment remained a fixed postoperative group after follow-up had begun | Not adequately addressed | Not adequately addressed |
| Feng et al, 2024 | Cox regression in selected pathological subgroups; included overall comparison was unadjusted | No aligned time zero, landmark analysis, or time-dependent exposure | Not adequately addressed | Not adequately addressed |
| Geng et al, 2026 | Stabilized inverse probability weighting | Ninety-day survival restriction partly addresses OS but does not explicitly remove pre-AIT recurrence for DFS | Partially addressed | Not adequately addressed |
| Huang et al, 2026 | Propensity-score matching and Cox regression | RFS began at surgery before postoperative treatment initiation | Not applicable | Not adequately addressed |
| Kelly et al, 2021/2025 | Randomization and stratified Cox analysis | Treatment assignment and follow-up began at randomization in both arms | Addressed | Addressed |
| Koga et al, 2025 | Multivariable Cox regression | Six-month landmark directly addressed DFS only; the original OS analysis remained surgery-based | Not adequately addressed | Partially addressed |
| Liu C et al, 2025 | Multivariable Cox regression; included KM comparison was unadjusted | No timing-specific method was reported | Not adequately addressed | Not adequately addressed |
| Park et al, 2022 | Randomization and Cox regression | Treatment assignment and follow-up began at randomization in both arms | Addressed | Addressed |
| Sakowitz et al, 2026 | Entropy balancing and multivariable Cox regression | Adjustment for 90-day morbidity does not remove guaranteed survival before AIT initiation | Not adequately addressed | Not applicable |
| Sugase et al, 2025 | Propensity-score matching and multivariable Cox regression | Three-month event-free restriction reduces early-event bias, but some treatment began after 10 weeks | Partially addressed | Partially addressed |
| Verhoeven et al, 2026 | Propensity-score trimming and nearest-neighbor matching | Matched pseudo-index dates aligned the untreated comparison with AIT initiation | Addressed | Not applicable |
| Wang et al, 2026 | Multivariable Cox regression | Both endpoints began at surgery and AIT initiation timing was not reported | Not adequately addressed | Not adequately addressed |

Abbreviations: AIT, adjuvant immunotherapy; DFS, disease-free survival; IQR, interquartile range; KM, Kaplan-Meier; NCDB, National Cancer Database; NR, not reported; OS, overall survival; RFS, recurrence-free survival. Addressed indicates that treatment assignment and survival time zero were aligned by randomization or a matched index date. Partially addressed indicates an endpoint-relevant early-event restriction or landmark analysis without a time-dependent exposure model. Not adequately addressed indicates that follow-up began before postoperative treatment assignment without an appropriate endpoint-specific landmark or aligned index date. Matching, weighting, entropy balancing, and conventional Cox adjustment were not considered sufficient by themselves. The magnitude of bias could not be quantified for most studies because individual treatment-start dates and events before treatment initiation were unavailable. When present, this bias would generally favor AIT. In Koga et al, the DFS HR attenuated from 0.23 to 0.40 after a six-month landmark analysis.
